# Measurement of public space justice in traditional villages with coexistence of old and new based on villagers’ perceptions

**DOI:** 10.1371/journal.pone.0352578

**Published:** 2026-07-07

**Authors:** Bingbin Tian, Yunyuan Deng, Mingcheng Jiang

**Affiliations:** 1 College of Geography and Tourism, Hengyang Normal University, Hengyang, Hunan, China; 2 National Local Joint Engineering Laboratory on Digital Preservation and Innovative Technologies for the Culture of Traditional Villages and Towns, Hengyang, Hunan, China; 3 Cooperative Innovation Center for Digitalization of Cultural Heritage in Traditional Villages and Towns, Hengyang, Hunan, China; International Islamic University Malaysia, MALAYSIA

## Abstract

Rural spatial justice studies lack frameworks addressing rural–urban contextual gaps. We bridge this by proposing a three-dimensional model (recognition-distribution-participation) to quantify villagers’ spatial justice perceptions in transitional villages with old-new coexistence. Using 314 surveys across Zhushan (Hunan) and Dabitou (Guangxi), structural equation modeling (SEM) confirms the model explains 63% of variance—highlighting the interconnected nature of recognition, distribution, and participation in shaping justice perceptions, a nuance that single-dimension approaches may overlook.Key results reveal: Distributive justice (e.g., infrastructure equity) dominantly shapes perceptions (β = 0.45);Recognition justice is mediated by cultural symbols but constrained by weak revitalization; Participatory justice remains critically low due to governance deficits. Qualitative contrasts suggest how endogenous governance in Zhushan may strengthen recognition, while capital-driven monopolies in Dabitou appear to suppress participation.This study advances rural geography by:Adapting urban-centered justice theory to rural transitions;Providing a preliminary metric for spatial equity assessment in similar aging rural contexts. Findings offer context-specific insights for optimizing public space allocation in transforming villages facing comparable demographic challenges.

## Introduction

During the deepening phase of China’s national rural revitalization strategy and urban-rural integration, rural public spaces play a crucial role in facilitating social connections, preserving cultural heritage and improving governance. Their reconstruction quality directly affects the internal vitality and fairness of rural revitalization. The 2025 Central Document No. 1, “Opinions on Further Deepening Rural Reform and Promoting Comprehensive Rural Revitalization,” calls for “exploring rural construction models with local characteristics,” emphasizing the protection of farmers’ spatial rights [1].However, as rural modernization accelerates, the “villages with coexisting old and new architectures” model, blending traditional and modern elements, is becoming a typical form of rural transformation in China. In these villages, traditional buildings and new modern structures coexist, which brings vitality, but also poses challenges to rural public space governance and cultural continuity.

Current research on rural public spaces reveals two major dilemmas. Firstly, there exists a disconnect between the material and cultural dimensions. While improvements to the living environment focus on hardware upgrades, the social function of public spaces as ‘sites of cultural memory’ is often neglected. For instance, Shang Village in Anhui revitalized traditional Dragon Boat Festival customs through its Bamboo Pavilion Hall, demonstrating that public spaces must host integrated ‘production-life-ritual’ activities to sustain collective memory [2]. Conversely, the Qing Dynasty architectural complex in Zhaibuchang Village risks becoming merely a ‘museum-style’ exhibit, failing to support villagers’ daily cultural practices.

Secondly, there is a paucity of agency in governance. Despite the fact that the majority of rural revitalisation strategies advocate ‘government guidance and community participation’, it is evident that villagers frequently adopt a passive role in practice. As Guo Qianqian observes, rural development initiatives often present a scenario in which ‘cadres deliver a monologue on stage while farmers observe from the audience below’, suggesting a degree of disregard for farmers’ agency [3]. In an analysis of rural agency in Yangshuo County, Guangxi, Zhang Lingyuan [4]contends that such agency is not a simple linear process of endogenous development. During the process of revitalisation, the failure to effectively convert ‘external impetus’ into internal momentum for rural societal growth results in insufficient positive interaction between rural collectives and individuals, leaving farmers’ agency inadequately realised. Xue Wenjing’s case study of the ‘Beautiful Countryside Conservation Project’ in Fanglin Village, S Province, revealed disparate levels of agency among farmers when interacting with external forces [5].

The present study posits that Spatial Justice Theory provides a robust analytical framework for understanding the aforementioned issues. Theorised originally in critical studies on the unequal distribution of urban spatial resources, this theoretical system has now reached a relatively mature stage of development. The theoretical foundation of socio-spatial dialectics was established by Henri Lefebvre [6–8]. In addition, Edward Soja’s ‘Thirdspace Theory’ profoundly elucidates spatial justice as the practical manifestation and contested terrain of social justice in the spatial dimension [9,10].David Harvey’s analysis, from a political economy perspective, reveals the uneven production and distribution of urban spatial resources under the logic of power and capital [11–13].Dadashpoor and Dehghan identified seven core categories of spatial justice (e.g., participation, power and governance, equality) through a systematic review, providing an integrated conceptual framework for understanding spatial justice [14].Luo,S and Luo,Z applied the Gini coefficient and a coupling coordination model to reveal significant spatial inequity and a declining coordination trend between ecosystem services and residents’ well-being in Jiangxi Province, China [15].

By contrast, research into spatial justice in rural areas emerged later and has had a relatively limited theoretical focus, with significant differences in context between urban and rural areas. Early domestic studies primarily focused on distributive justice concerning rural land resources. For example, Li Peilin revealed imbalances in the distribution of benefits caused by ambiguous property rights systems during rural land transfers [16], while Ye Xingqing emphasised that the equitable provision of public services, particularly infrastructure, constitutes a core dimension of rural spatial justice and advocated for closing the urban-rural gap [17].

Why Rural Spatial Justice Requires Distinct Theoretical Treatment. While spatial justice theory has been extensively developed in urban contexts—focusing on issues such as gentrification, transit equity, and the right to the city,its application to rural settings demands careful theoretical adaptation. Three structural differences between urban and rural contexts are particularly salient. First, demographic trajectories diverge sharply: rural areas in China and many other countries are characterized by depopulation, out-migration of youth, and accelerated aging [18], whereas urban areas face population concentration and diversification. These demographic patterns fundamentally shape who uses public space and whose justice claims are voiced. Second, the political economy of spatial production differs: rural public space transformation is frequently driven by tourism development and external capital injection rather than by endogenous population growth or market demand.This creates power asymmetries between villagers, developers, and local governments that are distinct from urban gentrification dynamics. Third, the socio-cultural function of public space varies: in rural communities, public spaces serve not only as sites of leisure and interaction but as repositories of collective memory, lineage identity, and ritual practice. The erosion of such spaces carries cultural consequences that differ qualitatively from the loss of urban public amenities. These distinctions necessitate a rural-specific operationalization of spatial justice—one that accounts for demographic vulnerability, external capital dominance, and the intertwining of spatial and cultural continuity.

In recent years, with the deepening practice of traditional village preservation and development, some scholars have begun examining spatial justice dilemmas therein. For example, Zhang Liang revealed how imbalanced power structures among “government-enterprise-villagers” in ancient village tourism development lead to cultural spaces being eroded by capital logic, squeezing indigenous cultural rights [19]; Yan Jiawei explored the reconstruction process of rural memory amid rapid transformation and its complex impact on villagers’ perception of public space justice [20].Jiaqi Bai divides the dimensions of tourism spatial justice, incorporates participatory justice and distributive justice into the traditional fairness preference theory, and verifies the perception model of tourism spatial justice from the perspective of community residents [21].

Nevertheless, extant research on rural spatial justice continues to demonstrate significant lacunae:Theoretical Adaptability Issues: It is imperative that core concepts originating from urban contexts (e.g., redistribution, participatory rights, recognition) are adapted theoretically and expanded conceptually within rural socio-cultural structures.

Critical Topic Deficiencies: The inherent contradiction between the imperative to “preserve traditional features and maintain cultural identity” and the need to “introduce modern facilities to meet development needs” — a contradiction that is particularly salient in transitional villages — has received scant attention from the academic community. This oversight is all the more lamentable given the significant implications it has for the concept of spatial justice.The existing literature on rural studies is predominantly focused on singular dimensions of analysis, such as distributive justice in facility equalization. This methodological approach fails to address the interrelated dimensions of recognition justice, distributive justice, and participatory justice, which are critical to a comprehensive understanding of the subject. Empirical measurement of how these dimensions synergistically shape villagers’ spatial justice perception – particularly their sense of agency – remains scarce.

**Fig 1:**
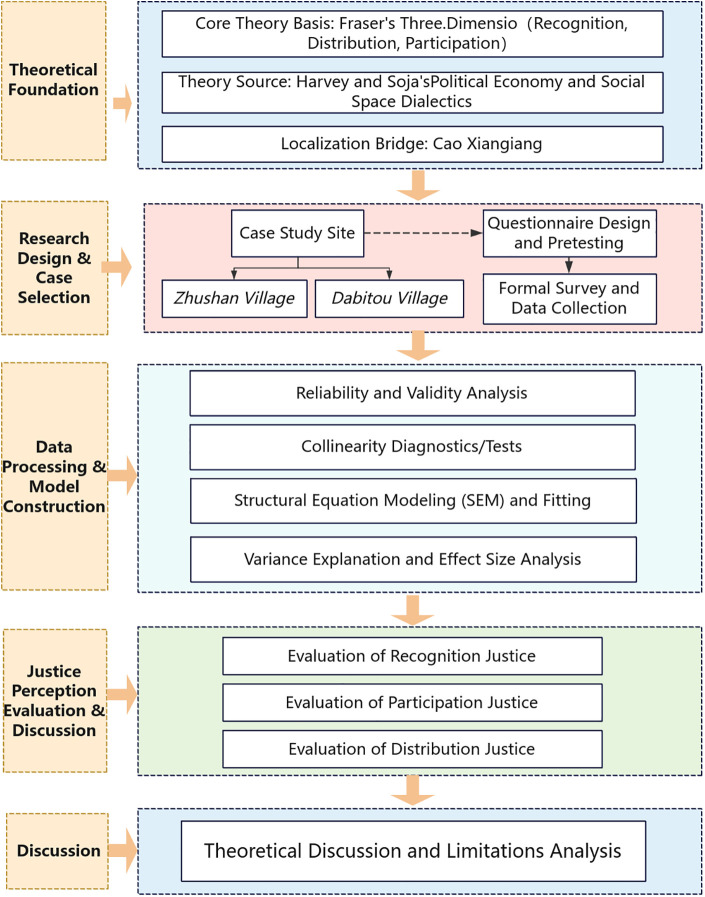
Technical Roadmap（Source: Self-drawn by the author).

Against this backdrop, the core contribution of this study lies in theoretical integration and innovation: The present study aims to build upon the systematic interpretation of spatial justice localization proposed by Cao Xianqiang and to integrate Nancy Fraser’s tripartite framework of recognition-redistribution-representation [22,23]. To this end, a three-dimensional justice perception model has been constructed, specifically for evaluating public spaces in transitional villages. The model is based on the synthesis of these two theoretical frameworks and is entitled the “Recognition-Distribution-Participation” model. This paper proposes a novel, empirically-derived index system to evaluate rural public space justice, with a focus on villager-centred approaches. The index system is developed and validated through rigorous empirical research, with case studies drawn from Hunan’s Zhushan Village and Guangxi’s Dabitou Village. This model overcomes the limitations of single-dimensional approaches by emphasising dynamic interrelations among the three dimensions, with the following objectives:To reveal concrete manifestations, causes, and interaction mechanisms of justice imbalances in public space governance;To provide justice-based scientific criteria and actionable pathways for optimising rural public space planning;To enhance governance efficacy while promoting cultural continuity and community integration;And ultimately, to facilitate high-quality rural revitalisation.As can be seen from Fig 1, the technical route of this study is as follows.

## Materials and methods

### Overview of the Study Area

**Fig 2:**
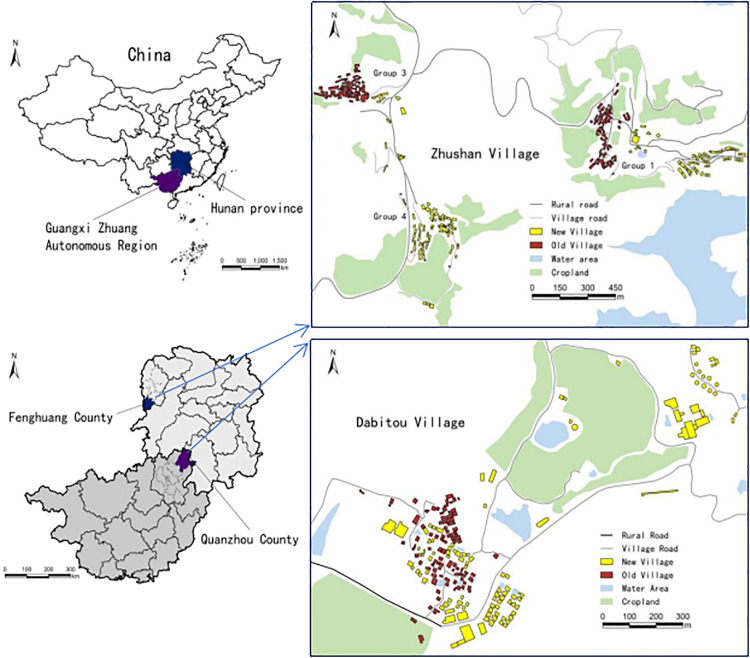
Location Map of Case Village（Source: Author’s own drawing using Natural Earth public domain data.).

This study examines two case sites: Zhushan Village in Hunan and Dabitou Village in Guangxi.As shown in Fig 2,both are typical traditional villages with coexisting old and new elements. Zhushan Village, with over 800 years of history, is known for its Miao culture and traditional architecture [24].Dabitou Village, blending Central Plains and Lingnan cultures, has preserved Ming – and Qing – dynasty architecture. The new village features modern homestays and wellness facilities [25].

**Fig 3:**
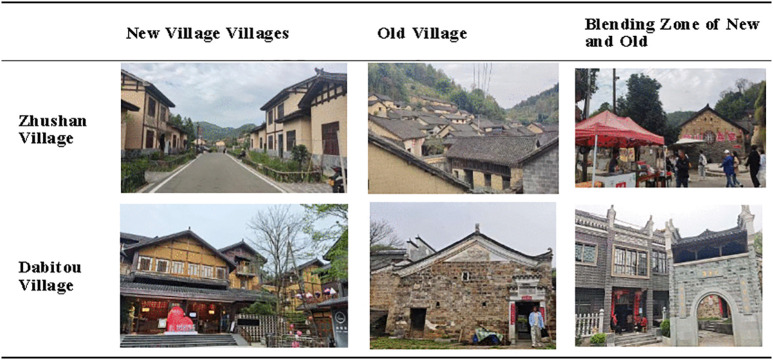
Comparison of Old and New Spatial Relationships in Case Villages(Source: Photo taken by the author.).

Zhushan and Dabitou are archetypal traditional settlements that exhibit a harmonious juxtaposition of historical and contemporary elements [26]. A new village has gradually developed on one side of the old village to preserve the integrity of the original architecture and ensure the continuity of traditional buildings. Tourism construction and new houses are located on flat, open land adjacent to the old village, as stipulated by tourism development policies. As villagers relocated, old courtyard houses were left vacant, creating a side-by-side pattern of old and new villages. This is especially pronounced in Zhushan Village, where one group has become a tourist attraction and group three plays a significant role. Most residents have moved to the new village, leading to significant hollowing out and gradual abandonment of the old village.As Fig 3 shows, it presents the old and new spaces of the case village.

**Fig 4:**
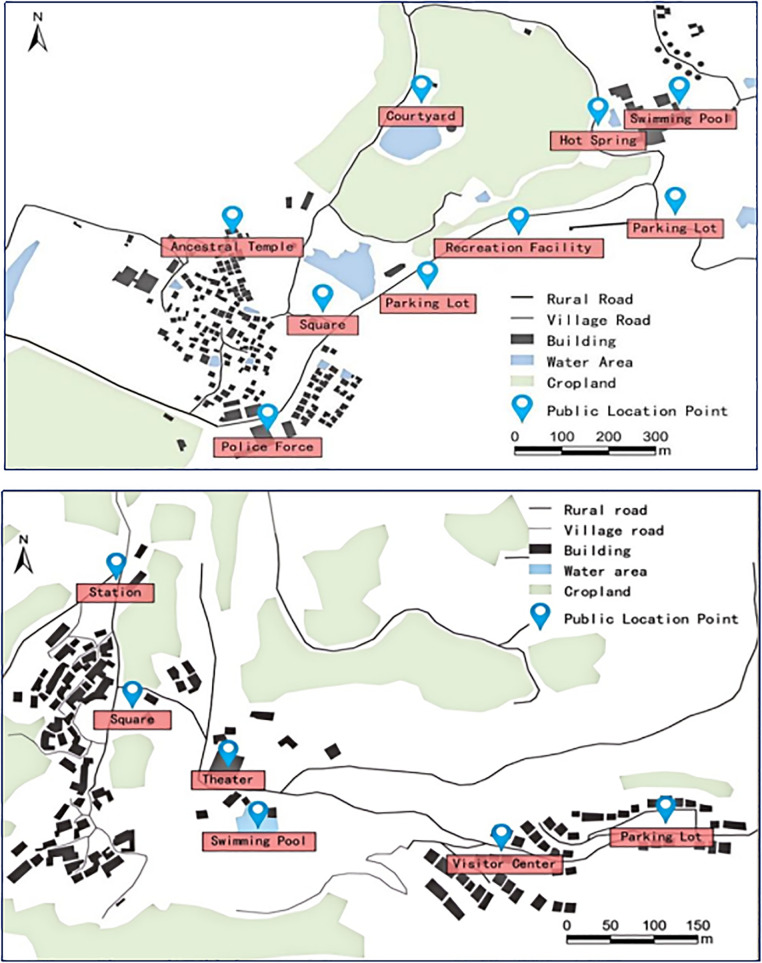
Main Public Space Areas of Group 1 in Zhushan Village (Nether)and Dabitou Village (Upper)（Source: Author’s own drawing using Natural Earth public domain data.).

Traditional-village public spaces—temples, stages, halls, wells, courtyards—are hybrid old/new arenas where villagers meet. Cao includes enterprises, rituals [27]; Dong adds places, authority, resources [28]. Here, the term denotes frequented sites for public, productive and social life: trees, wells, halls, fields plus the organised activities they host. Key survey points: Zhushan—entrance, scenic gate, car park, theatre plaza, shops; Dabitou—reception centre, old/new junction, Bin hall, new-village venues, car park [29].The main public spaces are illustrated in Fig 4.

### Questionnaire design and data collection

Based on Fraser’s social justice theory, this study operationalizes “participatory justice” within rural public space governance. The questionnaire was developed through a literature review and group discussions.An initial pool of 25 items was generated based on the theoretical framework and literature review. A pre-test was conducted with 30 villagers to assess clarity and relevance. Based on the pre-test results, 9 items were removed due to ambiguous wording, low item-total correlations, or cross-loading issues identified through exploratory factor analysis (EFA),the final instrument retained 16 items measured on a five-point Likert scale [30,31].

As summarized in Table 1, the final instrument comprised 16 items measured on a five-point Likert scale. While post-hoc analysis indicated that the removal of indicators P3 and D3 could yield a marginal increase in internal consistency, a decision was made to retain them. This decision was based on the established PLS-SEM guideline that loadings between 0.40 and 0.70 are acceptable in exploratory social science research when the indicator contributes substantively to the construct’s theoretical domain.

**Table 1:** Item screening and retention process.

Stage	Description	Numberof Items	Action Taken/ Rationale
1. Initial Pool Generation	tem s were generated based on Fraser’ s tripartite justice theory and a review of relevant rural spatial justice literature. The pool covered four dimensions: Recognition, Distribution, Participation, and Overall Perception.	25	
2. Pre-test & Item Purification	A pre-test was conducted with 30 villagers from a similar transitional village context. Data were subjected to Exploratory Factor Analysis (EFA) and reliability checks. Items with low communalities (<0.40), severe cross-loadings, or those that significantly improved Cronbach ‘s alpha upon deletion were flagged for removal.	25 → 16	9 items removed due to: (a) ambiguous wording leading to respondent confusion; (b) factor loadings below 0. 50; (c) high cross-loadings on unintended dimensions.
3. Final Instrument	Final instrument retained for main survey. All 16 items were included based on theoretical and empirical considerations after the pre-test purification.	16	All 16 items retained for main analysis. Marginal items were retained on theoretical grounds to preserve content validity and domain coverage of the Participation and Distribution dimensions. Sensitivity analyses confirmed that their removal did not alter the significance or direction of the structural paths.

The survey covered the three spatial justice dimensions—recognition, distribution, and participation—along with villagers’ demographic characteristics and perceptions of public space justice. It was administered via face-to-face, one-on-one interviews in Zhushan Village (October 1–6, 2024) and Dabitou Village (April 3–7, 2025), spanning 11 days in total. This method ensured a high questionnaire return rate and data authenticity. After excluding invalid responses, 314 valid questionnaires were obtained. The measurement items for each dimension are presented in Table 2. This study was reviewed and approved by the Research Ethics Committee of Hengyang Normal University (Approval No.2025LL16). Oral informed consent was obtained from all participants prior to data collection. The consent procedure, including the verbal script used by interviewers, was reviewed and approved as part of the ethics protocol. Given that the majority of respondents were middle-aged and elderly with limited formal education, written consent was deemed culturally inappropriate and potentially intimidating. Instead, interviewers read a standardized consent script in plain, accessible language that explained: (a) the purpose of the research; (b) the voluntary nature of participation; (c) the right to withdraw at any time without penalty; (d) the confidential handling of responses; and (e) the expected duration of the interview. Interviewers verbally confirmed comprehension by asking participants to restate the key points in their own words. For elderly participants (≥65 years, N = 62), interviewers paid particular attention to ensuring that respondents were alert, oriented, and able to engage in a coherent conversation about their village. In cases where a potential respondent exhibited confusion or an inability to understand the study’s purpose, the interview was not initiated. All consent outcomes were documented on field record sheets maintained by the two-person research team. For underage participants (N = 12), consent was obtained from accompanying parents or guardians, and the minors provided verbal assent.

**Table 2:** Items for each dimension.

Dimension	Code	Measurement Item
Recognition Justice	A1	Reflection and inheritance of local culture
	A2	Maintenance and renewal of public facilities|
	A3	Expansion of public space area
	A4	Conducting socio – cultural activities
Distribution Justice	D1	Reasonable construction of infrastructure
	D2	Construction of leisure and entertainment facilities
	D3	Environmental protection and waste sorting
	D4	Accessibility of village transportation
Participation Justice	P1	Consultation and response to public affairs
	P2	Transparency
	P3	Fair feedback
	P4	Sustained participation
Overall Perception	T1	Happiness
	T2	Satisfaction
	T3	Sense of fairness
	T4	Sense of identity

While the primary quantitative analysis focuses on the pooled sample to validate the theoretical model, illustrative qualitative case evidence from semi-structured interviews and field observations in Zhushan and Dabitou is used to contextualize and interpret the statistical findings, revealing potential context-specific mechanisms of justice perception.

### Analytical approach

Given the exploratory nature of this study, which aims to predict villagers’ overall perception of justice based on three interconnected dimensions, and considering the relatively moderate sample size (N = 314), Partial Least Squares Structural Equation Modeling (PLS-SEM) was adopted as the primary analytical technique. PLS-SEM is well-suited for prediction-oriented research and complex models where the theoretical foundation is still developing. All analyses were conducted using SmartPLS 3.0 software.

A total of 314 valid responses were collected, comprising 149 from Zhushan Village and 165 from Dabitou Village. Given the modest sub-sample sizes, formal multi-group analysis was not conducted; village-level comparisons in this study are therefore based on qualitative case evidence rather than statistical inference.

### Demographic characteristics analysis

It can be known from Table 3 that the sample had slightly more males (53.4%) than females (46.6%). The largest age group was 45–64 years old (45.4%). Most respondents had low education levels, with 65.2% having a primary school education or lower. Farming was the most common occupation (35.5%), followed by tourism service workers (18.8%).

**Table 3:** Descriptive statistics of sample information.

Variable	Specific Composition	Frequency	Percentage
Gender	Male	167	53.4
	Female	146	46.6
Age	Under 14	12	3.8
	15-24	18	5.8
	25-44	79	25.2
	45-64	142	45.4
	Over 65	62	19.8
Education Level	Primary School or Below	204	65.2
	Junior High School	61	19.5
	Vocational School or High School	41	13.1
	Associate or Bachelor’s Degree	6	1.9
	Master’s Degree or Above	1	0.3
Average Monthly Income	2000 or Below	191	61.0
	2000-5000	92	29.4
	5000-8000	24	7.7
	Over 10000	4	1.3
Occupation	Farming	111	35.5
	Tourism Service Worker	59	18.8
	Inn (Farmhouse) Owner	30	9.6
	Tourism Product Production, Processing	11	3.5
	Student	22	7.0
	Village Cadre	29	9.3
	Other	35	11.2

### Analysis and validation of the measurement model

#### Descriptive statistics and normality test of villagers’ justice perception scale.

The data distribution was assessed for normality using skewness and kurtosis statistics. Following conventional criteria, absolute values of skewness < 3 and kurtosis < 8 indicate approximate normal distribution [32]. As shown in the table below, all measurement items met these thresholds, confirming that the data satisfactorily approximate a normal distribution. This supports the robustness of subsequent statistical analyses of public space justice dimensions and their interrelationships.

### Reliability analysis

The Cronbach’s alpha coefficient was used to assess internal consistency.The 16-item instrument demonstrated satisfactory reliability. While post-hoc analysis suggested that removing items with comparatively lower loadings (P3, D3) could marginally improve internal consistency, we retained them to preserve the theoretical breadth and content validity of each dimension as initially conceptualized. Table 4 shows the final Cronbach’s alpha and CR values for all dimensions still exceed the recommended thresholds, confirming adequate reliability.

**Table 4:** Reliability statistics.

Dimension	Number of Items	Cronbach’s alpha
**Recognition Justice**	4	0.738
**Distribution Justice**	4	0.745
**Participation Justice**	4	0.797
**Overall Perception**	4	0.881
**Overall Scale**	16	0.863

### Validity analysis

The KMO value was 0.829, and the Bartlett’s test of sphericity was significant (p = 0.000), indicating suitability for factor analysis. Convergent and discriminant validity were assessed using Smart PLS 3.0. Results of Table 5 showed good internal consistency and convergent validity.

**Table 5:** KMO and Bartlett’s Test.

KMO Value		0.829
Bartlett’s Test of Sphericity	Bartlett’s Test of Sphericity	2061.619
	Degrees of Freedom (DF)	120
	Significance Level (p)	.000

Reliability refers to the internal consistency of a scale, while validity assesses whether items effectively measure their intended constructs. For validating scales with predefined dimensions, Confirmatory Factor Analysis (CFA) is appropriate [33]. Accordingly, we performed CFA using Smart PLS 3.0 to evaluate the measurement model, focusing on convergent and discriminant validity. As shown in Table 6, all composite reliability (CR) values exceeded 0.7, indicating good internal consistency, and all average variance extracted (AVE) values surpassed 0.5, confirming adequate convergent validity.

**Table 6:** Convergent Validity Test Results.

Dimension	Items	Factor Loading	AVE (Average Variance Extracted)	CR (Composite Reliability)
Recognition Justice	A1	0.78	0.62	0.88
	A2	0.72		
	A3	0.68		
	A4	0.75		
Distribution Justice	D1	0.76	0.58	0.85
	D2	0.69		
	D3	0.61		
	D4	0.65		
Participation Justice	P1	0.73	0.53	0.82
	P2	0.66		
	P3	0.53		
	P4	0.70		
Overall Justice Perception	T1	0.85	0.67	0.90
	T2	0.82		
	T3	0.74		
	T4	0.79		

Note: Following guidelines for PLS-SEM [34], indicator loadings above 0.70 are considered ideal, while loadings between 0.40 and 0.70 are acceptable for exploratory research if they do not compromise the construct’s composite reliability or average variance extracted. Indicators P3 (λ = 0.53) and D3 (λ = 0.61) were therefore retained on theoretical grounds to ensure adequate coverage of the respective justice dimensions.

Sensitivity Analysis for Indicator P3. The factor loading for indicator P3 (‘Fair feedback’) was 0.53, which falls below the conventionally recommended threshold of 0.60 for confirmatory research. To evaluate whether the retention of P3 materially affects our substantive conclusions, we conducted a sensitivity analysis by temporarily removing P3 from the measurement model and re-estimating the structural paths. The results are summarized below:The path coefficient from Participation Justice to Overall Perception changed marginally from β = 0.28 (p = 0.003) to β = 0.26 (p = 0.005), remaining statistically significant and substantively unchanged;The R² for Overall Perception decreased only slightly from 0.63 to 0.62;The composite reliability for Participation Justice improved from 0.82 to 0.85, and AVE increased from 0.53 to 0.58. Given that the core findings are robust to the exclusion of P3, we elected to retain this indicator in the final model. The retention decision was guided by theoretical considerations: P3 captures the perceived fairness of feedback mechanisms, a conceptually distinct facet of participation justice that is not fully covered by the other three indicators (consultation, transparency, and sustained participation). Removing P3 would narrow the content validity of the participation justice construct. We acknowledge the lower loading as a measurement limitation and recommend that future scale development efforts refine the wording of P3 to improve its psychometric performance.

As shown in the Table 6 above, the composite reliability (CR) values for all dimensions exceed 0.7, indicating good internal consistency. Additionally, the average variance extracted (AVE) values for all dimensions are greater than 0.5, demonstrating that the convergent validity has been achieved. This confirms that each item in the scale effectively contributes to the overall measurement of justice perception.

Note: The bold diagonal values represent the square root of the Average Variance Extracted (AVE) for each dimension. As shown in Table 7, the square roots of the AVE values for each dimension are presented on the diagonal. According to the Fornell-Larcker criterion, the correlations between different dimensions should be lower than the square root of their respective AVE value [34].Since all off – diagonal correlations are lower than the corresponding AVE square roots, this confirms that the dimensions are distinct and discriminant validity is established. This result enhances the reliability of the study.

**Table 7:** Discriminant Validity Test.

Dimension	Recognition Justice	Distribution Justice	Participation Justice	Overall Justice Perception
Recognition Justice	**0.79**			
Distribution Justice	0.45	**0.76**		
Participation Justice	0.38	0.31	**0.73**	
Overall Justice Perception	0.52	0.61	0.47	**0.82**

Note: The bold diagonal values represent the square root of the Average Variance Extracted (AVE) for each dimension.

### Multicollinearity test

After the measurement model has successfully passed the validation tests, it is essential to ensure that the structural equation model is not affected by multicollinearity among the predictor variables. To this end, the Variance Inflation Factor (VIF) is employed as a diagnostic tool for multicollinearity. This diagnostic process involves both the measurement model and the structural model to comprehensively assess multicollinearity issues. Generally, a VIF value less than 5 indicates that multicollinearity is not a concern [35].

As shown in Table 8, all VIF values for the predictor variables are below 5, indicating that multicollinearity is not a significant issue in the structural model. This ensures that the model’s estimates are reliable and valid for further analysis.

**Table 8:** Multicollinearity test for the structural model.

Predictor Variable	VIF Value
Recognition Justice	1.82
Distribution Justice	2.15
Participation Justice	1.75
Overall Justice Perception	3.14

### Variance explained and effect size test

The variance explained, denoted by R^2,^ measures the extent to which the independent variables account for the variation in the dependent variable. It ranges from 0 to 1, with higher values indicating stronger explanatory power. According to research by Chin and others, R^2^ values can be categorized into three levels of explanatory power: when R^2^ is between 0.190 and 0.330, the explanatory power is considered weak; when R^2^ is between 0.330 and 0.670, it is considered moderate [36]. An R^2^ value of 0.63 indicates that the model explains 63% of the variance in the dependent variable, which is considered a moderate level of explanatory power.

Based on Cohen’s criteria for effect size, f^2^ values less than 0.020 are considered weak; values between 0.020 and 0.15 are considered moderate; and values exceeding 0.260 are considered strong [37]. As shown in Table 9, R^2^ is only applicable to the dependent variable (overall perception), reflecting the model’s explanatory power regarding villagers’ overall justice perception (63%), which is at a moderate level. The f^2^ effect size measures the individual impact of each independent variable on the dependent variable: Distribution Justice (f^2^ = 0.25) has an effect close to a large effect, indicating its strongest marginal contribution to overall perception; Recognition Justice (f^2^ = 0.12) has a moderate effect, while Participation Justice (f^2^ = 0.08) has a small effect, consistent with Cohen’s (1988) effect size classification [37]. This further confirms the central role of Distribution Justice and the relatively weaker role of Participation Justice.

**Table 9:** Variance explained (R^2^) and Effect Size (f^2^).

Dimension	Variance Explained(R^2^)	Effect Size (f^2^)	Conclusion
Overall Justice Perception	0.63	–	
Recognition Justice	–	0.12	moderate effect size
Distribution Justice	–	0.25	moderate to large effect size
Participation Justice	–	0.08	small effect size

Additionally, the model’s predictive relevance was assessed using the Stone-Geisser’s Q² value.A blindfolding procedure was run in Smart PLS with an omission distance of 7.The resulting Q² value for the endogenous latent variable (Overall Justice Perception) was 0.625.This confirms the model’s predictive validity beyond merely explaining variance.

### Path analysis

Using the PLS algorithm in Smart PLS 3.0 software, the path coefficients were calculated, and their significance was tested using the Bootstrapping method. The number of bootstrap subsamples was set to 5,000, which is consistent with the standard practice in current PLS-SEM empirical research. Compared with the original results, the path coefficients remained unchanged, the standard errors decreased slightly, and the t-values increased marginally. All paths were significant at the p < 0.001 level, and all hypotheses were supported. If the absolute value of the t – statistic exceeds 1.96 and the p – value is less than 0.05, the path coefficient is considered significant [38,39]. As shown in Table 10, three paths were identified:

**Table 10:** Path coefficients and bootstrapping results.

Path Relationship	Standardized Coefficient	Standard Error	T Statistics	P.Value	Hypothesis Supported?
Recognition Justice → Overall Perception	0.32	0.05	6.70	<0.001	Supported
Distribution Justice → Overall Perception	0.45	0.04	11.80	<0.001	Supported
Participation Justice → Overall Perception	0.28	0.06	4.90	0.003	Supported

Distribution Justice → Overall Perception (β = 0.45): With the largest coefficient, this indicates that the fairness of resource distribution is the most core factor influencing overall perception. For every one – standard – deviation increase in distribution justice, the overall perception increases by 0.45 standard deviations, which is considered a moderately strong effect. The finding that “62% of villagers believe that improving roads is more practical than holding village meetings” reflects the survival rationality in rural governance, where material satisfaction takes precedence over procedural fairness (Interview record with villagers in Zhushan Village, 2024 − 10 − 03). The significant impact of distribution justice aligns with theoretical expectations, as the fair allocation of resources such as infrastructure and recreational facilities has the strongest effect on enhancing residents’ overall perception. This is likely because material resources directly affect the quality of life and are the most easily perceived dimension of justice by residents.

Recognition Justice → Overall Perception (β = 0.32): With the second – largest coefficient, the effect size is close to 70% of that of distribution justice. Recognition justice has a promoting effect, as “soft” recognition mechanisms such as cultural heritage and maintenance of public spaces enhance a sense of belonging, which indirectly improves overall perception. However, its effect is weaker than that of distribution justice.

Participation Justice → Overall Perception (β = 0.28): Although it has the smallest coefficient, it is still positively significant, indicating that individual participation in the decision – making process, such as opportunities for expressing opinions, has a relatively weak but non – negligible effect on overall perception, with an effect size of about 62% of that of distribution justice. The contribution of participation justice is limited. While channels for participation such as consultation and feedback are significant, their effects are small, which may be related to insufficient opportunities for residents to participate or the formalization of feedback mechanisms.

**Fig 5:**
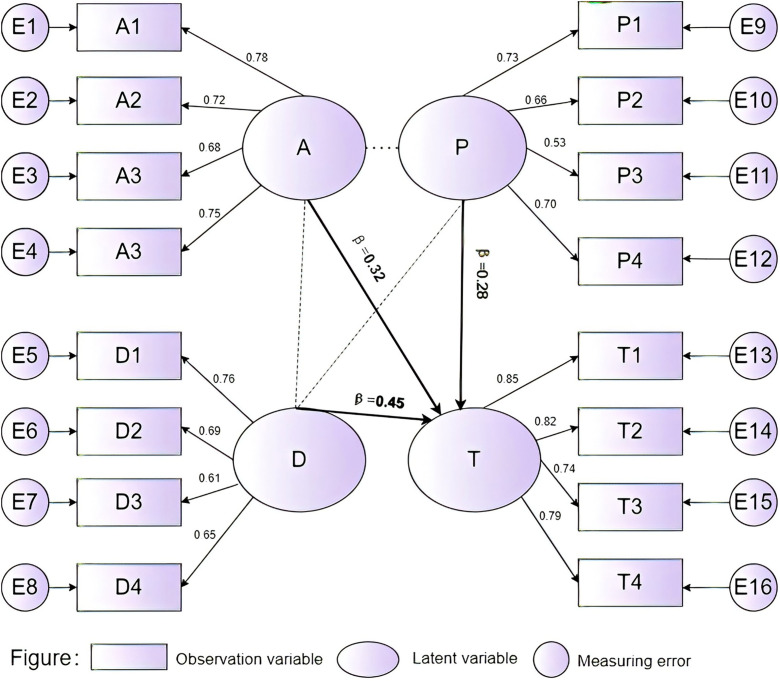
Structural Equation Model Diagram（Source: Self-drawn by the author）.

Note: Path coefficients are standardized (β). Significance levels are based on bootstrapping with 5000 subsamples.

Based on the above, the final structural equation model of public space justice perception is shown in the following Fig 5:

## Results

### Justice perception analysis and evaluation

This study used a structural equation model to validate the impact of four dimensions—Recognition Justice, Distribution Justice, Participation Justice, and Overall Perception—on villagers’ overall perception of justice in public spaces. The study found the following:

**(1) Recognition Justice:** The Dual Challenge of Cultural Heritage and Spatial Identity：Data from Table 11 show that cultural reflection and heritage are insufficient (A1 and A4 have medium scores), and villagers’ recognition of “social and cultural activities” (A4, M = 3.04) is relatively low. This reflects the insufficient embedding of traditional folk activities and local cultural symbols in public spaces in villages with coexisting old and new elements. For example, although Zhushan Village retains Miao architecture, the integration of modern tourism facilities (such as infinity pools and performance dramas) with traditional living spaces still has room for improvement. As a result, cultural heritage remains at the material level rather than as a living experience.

**Table 11:** Descriptive Statistics and Normality Test Results for Each Dimension.

Dimension	Measurement Item	M	SD	Skewness	Kurtosis	OverallM	OverallSD
Recognition Justice	A1 A2 A3 A4 D1	3.34 3.57 3.67 3.04 3.71	0.873 0.802 0.786 0.852 0.756	0.048 −0.113 −0.231 0.277 −0.366	−0.738 −0.434 −0.304 −0.565 0.191	3.405	0.828
Distribution Justice	D2 D3 D4	2.97 3.25 3.75	0.895 0.935 0.7	0.537 −0.137 −0.17	−0.474 −0.633 −0.11	3.420	0.822
Participation Justice	P1 P2 P3 P4 T1	2.47 2.58 2.43 2.18 3.61	0.8 0.817 0.798 0.798 0.726	0.657 0.578 0.751 1.033 0.131	0.801 0.643 1.36 2.024 −0.095	2.415	0.803
Overall Perception	T2 T3 T4	3.6 2.85 3.63	0.745 0.713 0.807	−0.372 0.179 −0.076	−0.128 0.001 −0.479	3.423	0.748

To address this issue, traditional public spaces such as ancestral halls, opera stages, and ancient wells in the old village can be transformed into “cultural memory nodes.” For instance, in Zhushan Village, a Miao ancestral hall could be equipped with intangible cultural heritage workshops (for silver – smithing and Miao embroidery experiences), and regular “Miao Festival Open Days” could be held, inviting villagers to participate in song and dance performances and handicraft teaching. This would make traditional spaces carriers for cultural heritage and villager interaction.

In the new village’s public spaces, such as squares and community centers, local cultural symbols can be embedded. For example, in Dabitou Village, the exterior walls of homestays could be painted with murals depicting the history of the Xiang – Gui ancient post road, and streetlights could be designed in the shape of Ming – and Qing – dynasty lanterns, creating a cultural narrative that changes with each step.

Moreover, the maintenance and updating of public facilities show inter – generational differences (A2 and A3 have higher scores). The scores for “maintenance and updating of public facilities” (A2, M = 3.57) and “expansion of public space area” (A3, M = 3.67) are relatively high, indicating that villagers recognize the material improvement of infrastructure. However, this may overlook the protective maintenance of traditional facilities such as ancient wells and stone – paved roads. For example, when Dabitou Village restored the ancient post road, if it only pursued modern functions such as drainage systems and ignored historical authenticity, it could lead to a rupture in traditional spatial memory.

To address this issue, Dabitou Village could establish a “classification maintenance mechanism for new and old facilities.” For traditional public spaces such as ancestral halls and opera stages, the principle of minimal intervention should be adopted to preserve historical traces as much as possible. For newly constructed facilities such as homestays and wellness areas, local materials such as wood and blue bricks could be integrated to achieve “formal symbiosis and functional complementarity.” Alternatively, in the public spaces of the new village, such as squares and community centers, local cultural symbols could be embedded. For example, the exterior walls of homestays could be painted with murals depicting the history of the Xiang – Gui ancient post road, and streetlights could be designed in the shape of Ming – and Qing – dynasty lanterns, creating a cultural narrative that changes with each step.

**(2) Distribution Justice:** The Contradiction Between Resource Balance and Spatial Fairness;The high scores for D1 and D4, and the lowest score for D2, indicate a “functional imbalance” in the allocation of infrastructure. “Transportation convenience” (D4, M = 3.75) and “rationality of infrastructure construction” (D1, M = 3.71) are highly rated, reflecting that the transportation network and basic guarantees such as water and electricity in traditional villages with coexisting old and new elements are relatively well – developed. However, the lowest score for “construction of leisure and entertainment facilities” (D2, M = 2.97) reveals that existing public spaces focus on practicality but neglect experience. For example, new villages may be equipped with parking lots and public restrooms but lack squares and pavilions suitable for daily interaction among villagers, resulting in a lack of vitality in public spaces.

To address this issue, a “demand - oriented facility configuration” strategy can be followed. Villagers can be involved in participatory planning through seminars and questionnaires to identify the core needs of different age groups. For example, if the majority are middle – aged and elderly, priority can be given to building social spaces such as senior activity centers and cultural opera stages. Balancing the density of facilities in new and old areas can prevent the polarization of “facility surplus in new villages and service deficiency in old villages.”

Regarding the challenge of environmental protection and sustainability, the medium score for D3 (M = 3.25) is associated with that villagers’ perception of ecological governance has not yet reached the expected level. Traditional villages with coexisting old and new elements often introduce external elements due to tourism development. However, without unified environmental governance standards, the ecological burden on old villages may increase. For example, the edge areas between new and old villages may have insufficient waste – handling capacity, and the public space environment in the gray vacuum areas is significantly worse than that in new villages. It is recommended to implement “coordinated environmental governance for new and old areas.” In the core area of the old village, “micro - renovation” can be carried out, introducing ecological treatment technologies such as artificial wetlands for domestic sewage treatment. In the new village, an ecological buffer zone can be designated to restrict excessive commercial development and ensure inter – generational fairness in the allocation of environmental resources.

**(3) Participation Justice:** The Absence of Villager Subjectivity and Governance Mechanism Shortcomings:The table shows that the accessibility of public affairs participation channels is insufficient (P1 - P4 have generally low scores). The indicators “consultation and response to public affairs” (P1, M = 2.47) and “sustained participation” (P4, M = 2.18) have the lowest scores, reflecting that villagers are mostly “passive recipients” rather than “active participants” in public space governance. As reflected in interviews with Dabitou villagers, ‘We farmers can’t participate in their tourism development. Even if new houses are built, we can’t enjoy the benefits.’ (Interview record, Dabitou, 2025-04-05).This sentiment aligns with the quantitative finding that Participation Justice has the lowest mean score (M = 2.415) across the entire sample, and qualitative case comparisons suggest this deficit may be particularly acute in capital-driven development contexts like Dabitou.

The low scores for “transparency” (P2, M = 2.58) and “fair feedback” (P3, M = 2.43) suggest that villagers have insufficient trust in the governance process. This is consistent with the interviews in Dabitou Village, where “land - transfer compensation standards are dominated by enterprises, and the village committee lacks negotiation ability,” confirming the institutional absence of villager participation channels under external capital dominance. In the villager representative meetings dominated by enterprises, 70% of the decision – making items were not substantially discussed by villagers. Formal participation led to significantly low perception of participation justice.

In Dabitou Village, tourism development is dominated by the Bin family. Bin Enxin is the project investor, who has formed a “corporate - led + government - assisted” development pattern through a 50 – billion – yuan capital injection and a government – enterprise cooperation model. However, the excessive power of the family – run enterprise may lead to the hollowing – out of grassroots governance, making it difficult for villagers’ demands to be effectively expressed through the village committee. For example, the “village bully” phenomenon reflected by villagers may stem from the monopoly of key links such as resource allocation and land transfer by enterprises or related parties. The “rural revitalization service team” led by party building has not fully absorbed public opinion. In the tourism development of Dabitou Village, a large amount of collective land transfer is involved. However, villagers generally reflect that the compensation standard is low and lacks a dynamic adjustment mechanism. Some villagers’ land is rented at less than 300 yuan per mu for a long term, while enterprises obtain high profits through the development of hot springs, homestays and other projects, forming a situation where “enterprises eat meat and villagers drink soup.” This profit – distribution pattern has led to villagers’ resistance to development. Although the development project has increased the income of some villagers through land transfer, employment placement and dividend models, whether the land – expropriation compensation process is fair and whether the profit – distribution is transparent has caused many disputes. In addition, villagers’ participation in planning and decision – making is limited. For example, the transformation of residential styles and industrial layout are mostly dominated by enterprises, which marginalizes the interests of vulnerable groups. Traditional villages with coexisting old and new elements often face the interest game between external capital, such as tourism developers and local villagers. If there is a lack of standardized consultation mechanisms, it may lead to the tilt of spatial resources towards commercial use. For example, priority is given to building tourist facilities rather than public services for villagers, which weakens villagers’ sense of fairness. Therefore, it is necessary to build a multi – stakeholder governance network and establish organizations such as villagers’ deliberative councils and village – worthy councils. In the planning stage, “participatory design workshops” should be introduced to allow villagers to participate in the functional layout and rule – making of public spaces. A transparent feedback mechanism should be established, such as a village – affairs – disclosure platform and regular hearings, to ensure procedural justice in the governance process. A “profit - sharing and risk - sharing” mechanism should be established to clarify the “villager - participation - ratio” in public space development. For example, tourism profits should be used to support public facility construction. A third – party supervision institution, such as a villagers’ representative + expert committee, should be established to ensure the transparency of the resource – allocation process and avoid the unjust phenomenon of “a few people making decisions and the majority paying the bill.”

**(4) Overall Perception:** The Path to Enhancing Happiness and Identification:The study shows that “sense of fairness” (T3, M = 2.85) is the weak link in overall perception, indicating that villagers’ subjective experience of spatial justice is still limited by uneven distribution and lack of participation.Combining the goals of public space governance in traditional villages with coexisting old and new elements, justice perception can be strengthened from the following aspects: Material Space Level: Promote functional integration by “sewing together new and old spaces,” such as connecting new and old areas with landscape walkways and shared public service centers to avoid spatial fragmentation. Socio – Cultural Level: Rely on traditional public spaces like ancestral halls and opera stages to cultivate community memory. In new villages, embed local cultural symbols such as murals and intangible cultural heritage displays to enhance cross – generational cultural identification.Governance Mechanism Level: Establish a “dynamic feedback - continuous improvement” governance cycle. Regularly assess villagers’ perception data, such as the measurement model in this study, and optimize the supply of public spaces in a targeted manner. Achieve a governance transformation from “government - led” to “villager - empowered.”

Furthermore, the core element of implementing the rural revitalization strategy is the revitalization of talent. With the continuous advancement of urbanization, many young and middle – aged people with certain knowledge and cultural literacy no longer confine themselves to small towns but gradually migrate to first – and second – tier cities. From the interviews with residents in Zhushan Village and Dabitou Village, it is understood that most of the permanent villagers are over 50 years old. The construction and innovation of villages face the dilemma of insufficient intellectual support, leading to a “generational gap” in rural areas. Due to limited knowledge and advanced age, the villagers who stay in the ancient villages mostly cannot participate or lack the willingness to participate in the protection and development of traditional villages. The current “hollowing - out” and “aging” of rural talent have led to the “hollowing - out” of village culture and a “stagnant” situation in village construction and governance. The village committee, as the responsible body of the village, mainly cooperates with the government to carry out village protection and development work. Overall, its working mode often follows the methods of predecessors and lacks innovative consciousness.

**Fig 6:**
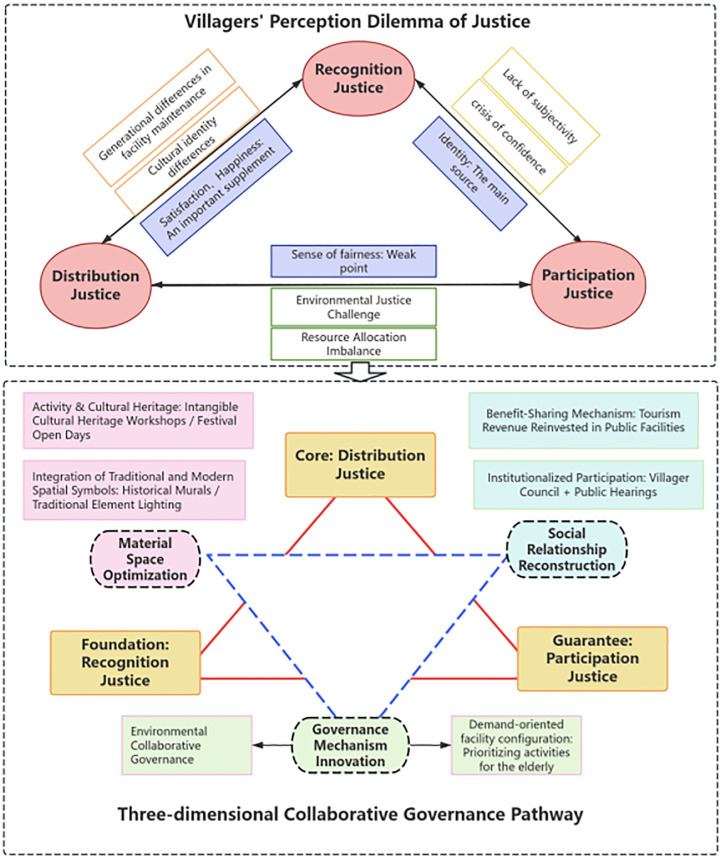
Analysis of the Results of justice Perception.

The development of Zhushan Village and Dabitou Village, like the development of most traditional villages with coexisting old and new elements, faces the problem of insufficient talent supply. Rural revitalization cannot be separated from the revitalization of traditional villages. The basis of village revitalization lies in ensuring talent revitalization. At present, the policy incentive mechanism for attracting talent backflow in Zhushan Village and Dabitou Village is not yet perfect, and there are many shortcomings in village protection and development. Relying solely on the “encircling - land” protection method cannot truly achieve the effective protection and development of Zhushan Village and Dabitou Village. The villagers’ justice perception dilemma is simply illustrated as shown in Fig 6.

### Limitations and future research directions

The RDP model (Recognition-Distribution-Participation) developed in this study collectively explains 63% of the total variance in villagers’ spatial justice perceptions, demonstrating its efficacy in assessing public space equity in transitional villages. This model not only provides a replicable justice evaluation tool for similar contexts but, more importantly, reveals a distinct constitution of spatial justice in rural settings: the influence of Distribution Justice (β = 0.45) significantly surpasses that of Participation Justice. This finding contrasts sharply with the higher demand for “participatory governance” often expressed by urban residents, highlighting how the urban-rural contextual divide reshapes the theoretical connotations of spatial justice. It offers a crucial empirical basis for advancing spatial justice theory within the urban-rural binary framework.

The theoretical contribution of this study lies in its successful adaptation of the spatial justice paradigm, originally rooted in urban critical theory, to the context of China’s rural transformation. By employing Fraser’s tripartite framework, it develops a structured methodology for assessing place-based justice. The integration of recognition, distribution, and participation into a unified index expands the purview of rural geography beyond traditional boundaries like land use efficiency, encompassing a broader spectrum of issues related to everyday justice. The results indicate that in rural areas with relatively scarce spatial resources, the equitable allocation of material resources such as infrastructure and public services is paramount to livelihoods. Inequitable distribution directly threatens well-being, establishing Distribution Justice as the primary ‘first-order condition’. While Recognition and Participation Justice remain significant, they function, to some extent, as ‘second-order conditions’, whose benefits are more fully realized only after basic material security is assured. Building on the empirical findings of the RDP model, we offer an exploratory and illustrative formulation of a Spatial Justice Index (SJI) for the studied villages: SJI = 0.45 × Distributive Justice + 0.32 × Recognition Justice + 0.28 × Participatory Justice. We emphasize that this formulation is statistically derived from a single sample and should not be interpreted as a generalizable policy weight. Path coefficients from PLS-SEM reflect the relative predictive importance of constructs within a specific model and sample; they are not designed to serve as normative weights for policy evaluation. This index is presented solely to illustrate how empirical findings might inform the conceptual development of future assessment tools, pending rigorous cross-validation and sensitivity testing. Any practical application of this index would require independent validation in new samples and explicit justification of weighting schemes beyond the empirical coefficients reported here.

With these important caveats in mind, the proposed formulation suggests several illustrative directions for rural planning and policy evaluation:

**As a Heuristic for Planning Comparison:** The index structure highlights the relative salience of distributive, recognition, and participatory concerns in shaping villagers’ justice perceptions. In planning scheme evaluations, this framework could serve as a conceptual checklist to ensure that all three dimensions are considered, with particular attention to distributive equity given its empirical prominence in this study.

**As a Framework for Policy Diagnosis:** The index offers a diagnostic lens for assessing whether specific policies (e.g., rural revitalization support, ecological compensation) address the justice dimensions that matter most to villagers. Low scores on participation items, for instance, may signal governance deficits requiring targeted intervention.

**As a Context-Sensitive Template:** Rather than prescribing fixed weights, the three-dimensional structure provides a transferable analytical template that can be populated with locally relevant indicators and recalibrated weights based on context-specific empirical work. Researchers and practitioners in other settings are encouraged to derive their own weighting schemes through comparable modeling efforts.

In summary, the SJI formulation offered here should be understood as a context-specific, sample-dependent illustration rather than a validated operational tool. Its primary value lies in demonstrating how empirical PLS-SEM results can be translated into a weighted multidimensional framework—a translation that must be undertaken anew for each distinct research or policy context.

This study has several limitations that warrant careful consideration when interpreting the findings.

Sample Representativeness and Age Bias. A significant limitation of this study is the pronounced age bias in the sample. Respondents aged 45 and above constitute 65.2% of the sample (45–64 years: 45.4%; 65 years and above: 19.8%), while individuals under 25 represent only 9.6% (under 14: 3.8%; 15–24: 5.8%). This distribution, while reflective of the demographic realities of rural hollowing, out-migration of youth, and population aging in contemporary China, severely limits the generalizability of our findings. The perceptions of spatial justice captured in this study are predominantly those of middle-aged and elderly, low-education rural residents. Younger villagers, migrant workers, and return migrants—groups whose perspectives on participation justice, cultural recognition, and aspirations for rural development may differ substantially—are underrepresented. Consequently, the observed weak effect of Participation Justice (β = 0.28) might be partially attributable to this demographic skew; younger, more educated villagers might place higher value on participatory mechanisms and voice in governance. We therefore caution against generalizing these findings to rural populations with different age structures or to younger demographic cohorts. Future research should employ stratified sampling strategies to ensure adequate representation of youth and should explicitly explore potential generational differences in justice perceptions through multi-group analysis or age-based moderation tests.

Reliance on Perceptual Survey Data. The research primarily relied on self-reported perceptual survey data collected through face-to-face interviews. While this approach ensures high response rates and data completeness, it is subject to common method bias and may not fully capture objective dimensions of spatial justice (e.g., actual accessibility, observable quality of facilities). Future studies could triangulate findings by incorporating objective methods such as spatial behavior observation, GIS-based accessibility analysis, or systematic audits of public space quality.

Social Desirability Bias. The use of face-to-face interviews, particularly for items related to governance quality and participation justice (e.g., P1–P4), introduces the potential for social desirability bias. Respondents might have been reluctant to express negative views about village leadership, development projects, or decision-making processes in the presence of researchers perceived as affiliated with external institutions. Several mitigation strategies were employed: (a) interviewers emphasized the academic, non-governmental nature of the study and assured respondents of complete anonymity; (b) questions were phrased neutrally to avoid leading respondents toward particular answers; (c) interviewers were trained to maintain a non-judgmental demeanor and to allow respondents to elaborate freely; and (d) the research team included local university students familiar with the dialect and cultural context, which helped establish rapport and reduce perceived power distance. Despite these measures, we acknowledge that the reported levels of participation justice and overall fairness perception may be overestimated relative to respondents’ private sentiments. Future research could complement face-to-face surveys with anonymous self-administered modes or indirect questioning techniques to better estimate the magnitude of social desirability effects in rural governance research.

Based on these limitations, future research should proceed in the following directions:

Deepening the Intergenerational Perspective: Given the intergenerational conflicts over resource allocation against the backdrop of rural “hollowing-out” (e.g., elderly villagers’ attachment to the old village versus younger villagers’ demand for new village development), future studies could explore constructing a four-dimensional rural spatial justice model incorporating “Intergenerational Justice.” This would systematically examine intergenerational equity through sub-dimensions like material resource allocation, cultural heritage participation, and access to development opportunities. Corresponding practical pathways include establishing “intergenerational dialogue platforms,” encouraging young villagers to learn traditional skills and organize cultural activities, while creating cultural heritage-related employment opportunities for elderly villagers, fostering intergenerational resource complementarity and cultural sharing.

Expanding Methodological Boundaries: Employ oversampling of younger groups via digital platforms (e.g., WeChat, Douyin) to address sample bias. Simultaneously, integrate “spatial behavior observation” with perceptual data to analyze the intrinsic relationship between public space usage efficiency and justice perceptions.

Enhancing Comparative Research: Investigate the impact of different governance models (e.g., collective self-management, government-enterprise-villager cooperation) on spatial justice perceptions across a wider range of case types, thereby refining more generalizable governance optimization pathways.

In summary, this study establishes and validates the RDP model, providing a robust theoretical framework and empirical tool for understanding and assessing spatial justice in transitional Chinese villages. The revealed imbalance among justice dimensions, coupled with the exploration of policy applications for the SJI, aims to bridge academic insights with planning practice, ultimately providing scientific support for promoting high-quality rural revitalization that is equitable and inclusive.

## Supporting information

S1 Dataset The original and sorted research basic data used in this paper. (XLSX)
