# Supplementary material for: Measurement of public space justice in traditional villages with coexistence of old and new based on villagers’ perceptions
Source: PLoS One. 2026 Jul 7;21(7):e0352578. doi: 10.1371/journal.pone.0352578 (PMC13340821; doi:10.1371/journal.pone.0352578)
Supplement: Attachment — Submitted filename: Response to Reviewers.docx [file pone.0352578.s002.docx]

## Response to Reviewers

**Manuscript ID:** PONE-D-25-67060

**Title:** Measurement of Public Space Justice in Traditional Villages with Coexistence of Old and New Based on Villagers' Perceptions

Dear Dr. Jamil Afzal and Reviewers,

We would like to express our sincere gratitude for the time and effort you have dedicated to reviewing our manuscript. Your insightful comments and constructive suggestions have been invaluable in helping us significantly improve the rigor, clarity, and overall quality of our work.

We have carefully considered all points raised by the reviewers and the editorial office. The manuscript has been thoroughly revised accordingly. Below, we provide a detailed, point-by-point response to each comment. For clarity, the reviewers' original comments are presented in italics, followed by our response and a description of the changes made. All line and page numbers referenced correspond to the revised manuscript with track changes (unless otherwise noted as the clean version).

**Responses to Reviewer #1**

Reviewer's Summary: The manuscript is partly technically sound... several internal inconsistencies and gaps in evidence weaken confidence that the conclusions are fully supported by the reported analyses.

Response: We thank the reviewer for this thorough and constructive assessment. We have carefully addressed each of the specific points raised, as detailed below.

Comment 1: The statistical methodology is not reported consistently. While the manuscript describes the use of SmartPLS and the PLS algorithm with bootstrapping, it simultaneously reports CB-SEM-style global fit indices... The authors should explicitly state whether the primary analytical framework is PLS-SEM or CB-SEM and report diagnostics appropriate to that choice.

Response: We fully acknowledge this critical inconsistency in the original submission. We have taken the following steps to rectify this issue:

(1) Clarified Analytical Framework: We have added a dedicated subsection titled "Analytical Approach" in the Materials and methods section (Page12, Line 359-368). This section explicitly states that Partial Least Squares Structural Equation Modeling (PLS-SEM) was the sole analytical framework employed, justified by the study's prediction-oriented nature and moderate sample size. We confirm that all analyses were conducted using Smart PLS 3.0.
(2) Removed Inappropriate Fit Indices: We have completely deleted the former Table 11 (Structural Model Fit Indices) and all associated text that reported CB-SEM metrics such as χ²/df, CFI, TLI, and RMSEA. These indices are not appropriate for variance-based PLS-SEM.
(3) Corrected Terminology: In the Path Analysis section and in Table 12, we have replaced the CB-SEM term "C.R. Value" with the PLS-SEM standard "T Statistics".
(4) Added PLS-SEM Specific Metrics: We have supplemented the model evaluation with the Stone-Geisser's Q² value to assess predictive relevance. As reported in the Variance Explained and Effect Size Test section (Page 18, Line 525), the blindfolding procedure yielded a Q² value of 0.625, confirming the model's predictive validity.
(5) Corrected Acknowledgments: We have removed the reference to AMOS from the Acknowledgments section to ensure consistency with the sole use of Smart PLS.

Comment 2: There is a key inconsistency between the abstract and the results section. The abstract reports the effect of distributive justice as β = 0.58, whereas the structural model table reports β = 0.45...

Response: We apologize for this typographical error. The correct standardized path coefficient is β = 0.45. We have corrected this throughout the entire manuscript, including the Abstract (Page 1, Line15), to ensure consistency with the final model output presented in Table 12.

Comment 3: The claim that the proposed framework "outperforms traditional one-dimensional models" is not currently demonstrated...

Response: We agree with the reviewer that the original phrasing was not supported by a formal model comparison. Following the reviewer's guidance, we have removed all claims of "outperforming" one-dimensional models. In the revised Abstract (Page 1, Line 10]), the text now emphasizes the value of the multidimensional framework in capturing the interconnected nature of justice perceptions, stating: "...highlighting the interconnected nature of recognition, distribution, and participation in shaping justice perceptions, a nuance that single-dimension approaches may overlook." We believe this more accurately reflects the contribution of our study without overstating the empirical evidence.

Comment 4: The manuscript's claims of "important spatial differences" between villages are not adequately substantiated with explicit quantitative testing... Either conduct multi-group analysis or reframe as qualitative observations.

Response: This is an excellent point. We have clarified that our discussion of village-specific dynamics is based on qualitative case evidence rather than statistical multi-group analysis. The following revisions were made:

(1) Methodological Clarification: In the Analytical Approach section (Page 12, Line 369-374), we have added a statement: "A total of 314 valid responses were collected, comprising 149 from Zhushan Village and 165 from Dabitou Village. Given the modest sub-sample sizes, formal multi-group analysis was not conducted; village-level comparisons in this study are therefore based on qualitative case evidence rather than statistical inference."
(2) Tempered Language: We have revised the language throughout the Abstract and Results sections to clearly frame spatial contrasts as qualitative observations. For example, the Abstract now states: "Qualitative contrasts suggest how endogenous governance in Zhushan may strengthen recognition, while capital-driven monopolies in Dabitou appear to suppress participation." In the Results section (Page 1, Line 17-20), we anchor these observations in interview excerpts and explicitly note that they are illustrative case contrasts.

Comment 5: Measurement development is insufficiently transparent and contains contradictions... provide a clear item-retention flow... justify the retention of lower-loading indicators.

Response: We have thoroughly revised this section for transparency and accuracy.

1. Item-Retention Flow: We have added a new Table 2 (Item Screening and Retention Process) in the Questionnaire Design section (Page 10), which clearly outlines the three stages from the initial 25-item pool to the final 16-item instrument.
2. Contradiction Removed: The contradictory sentence "After removing two items..." has been removed. The text now clearly states that 9 items were removed during the pre-test, resulting in the final 16 items.
3. Justification for Lower Loadings: We thank the reviewer for this important methodological observation. We have addressed this concern through both empirical sensitivity analysis and transparent theoretical justification, as detailed below:1.Sensitivity Analysis. We re-estimated the structural model after temporarily removing P3 from the participation justice construct. The key results are summarized in the table below (reproduced from our response to Reviewer #1, Comment 5, for ease of reference):The analysis demonstrates that removing P3 results in only marginal changes to the path coefficient (β = 0.28 → 0.26, p remains <0.01) and model fit (R² = 0.63 → 0.62). The substantive conclusions of the study are unchanged.

| **指标** | **保留P3的原始模型** | **删除P3后的模型** | **变化** |
| --- | --- | --- | --- |
| Participation Justice的CR | 0.82 | 0.85 | +0.03 |
| Participation Justice的AVE | 0.53 | 0.58 | +0.05 |
| 路径系数：Participation → Overall Perception | 0.28 | 0.26 | -0.02 |
| 该路径的T Statistics | 4.67 | 4.21 | 仍显著(p<0.001) |
| 该路径的P Value | 0.003 | 0.005 | 仍<0.01 |
| Overall Perception的R² | 0.63 | 0.62 | -0.01 |
| Participation Justice的f^2^ | 0.08 | 0.07 | -0.01 |

1. Justification for Retention. Based on the robustness of the findings and the theoretical importance of “fair feedback” to the participation justice construct, we elected to retain P3. This decision follows established PLS-SEM guidelines indicating that loadings between 0.40 and 0.70 are acceptable in exploratory social science research when the indicator contributes substantively to the construct’s theoretical domain and its removal does not compromise composite reliability or average variance extracted.
2. Acknowledgment of Limitation and Path Forward. We acknowledge that the loading of P3 (0.53) remains below the conventional threshold of 0.60. While our sensitivity analysis confirms that its removal does not alter the substantive conclusions, future scale development efforts should refine or replace this item (e.g., by rewording “fair feedback” more concretely or splitting it into multiple items) to improve psychometric robustness. We have noted this limitation in the revised manuscript (Validity Analysis section, Page 15, Line 426-435) and again in the Limitations section (Page 31, Line 998-1002) to guide future research.

Comment 6: The proposed Spatial Justice Index (SJI) weighting scheme appears potentially circular... should be presented as context- and sample-specific rather than generalized.

Response: We fully concur with this insightful methodological critique. We have significantly revised the presentation of the SJI in the Limitations and Future Research Directions section (Page 30, Line 888). The formula is now described as an "exploratory and illustrative formulation" and a "context-specific, sample-dependent illustration." We have added strong caveats stating: "We emphasize that this formulation is statistically derived from a single sample and should not be interpreted as a generalizable policy weight... This index is presented solely to illustrate how empirical findings might inform the conceptual development of future assessment tools, pending rigorous cross-validation and sensitivity testing." We have also removed language that framed it as an "operational tool."

Comment 7: Several additional points... bootstrapping with 1,000 subsamples... policy thresholds... limitations...

Response: We have addressed each of these points as follows:

(1) Bootstrapping: We have re-run the analysis with 5,000 subsamples (previously 1,000). The updated T Statistics and P Values are reported in Table 12 and referenced in the Path Analysis section (Page19,Line 547).
(2) Policy Thresholds: We have removed the specific, unsupported numeric thresholds (e.g., "30% attendance") from the Limitations section. The text now speaks in terms of general principles and benchmarks that would require further empirical calibration.
(3) Limitations and Language: We have significantly expanded the Limitations section (Page30, Line 939) to explicitly discuss the age bias in the sample and the potential for social desirability bias. We have also performed a full pass of the manuscript to temper causal language, replacing terms like "demonstrates" with "suggests" or "indicates" where appropriate.

**Responses to Reviewer #2**

Comment 1: Spatial Justice Index (SJI) derivation is statistically invalid -- Path coefficients cannot be directly used as policy weights. Remove the SJI formula or clearly state it is illustrative only, with proper justification.

Response: As noted in our response to Reviewer #1 (Comment 6), we have thoroughly revised the SJI section. We have retained the formula solely for illustrative purposes and have added explicit, strong caveats that it is not a validated policy weight and is specific to this sample. We hope this addresses the reviewer's statistical validity concern.

Comment 2: Severe age bias undermines generalizability -- Only 9.6% of respondents are under 25. Temper claims about global scalability and frame findings as specific to aging, low-education rural populations.

Response: We agree. We have taken the following actions:

(1) Revised Abstract and Conclusion: We have removed claims of global scalability. The Abstract (Page1,L22) now describes the metric as "preliminary" and relevant for "similar aging rural contexts."
(2) Expanded Limitations: We have added a dedicated subsection titled "Sample Representativeness and Age Bias" in the Limitations section (Page30,L939). This section details the age distribution, discusses the underrepresentation of youth, and explicitly cautions against generalizing the findings to younger or demographically different populations.
(3) Tempered language in the Author summary: We have further tempered the language in the Author summary by adding an explicit applicability caveat. Specifically, we now state that the proposed tool “is most directly applicable to aging, depopulating rural contexts and should be validated in younger populations before broader generalization.” This change ensures that the summary accurately reflects the sample limitations and avoids overstatement of generalizability.

Comment 3: Village comparison lacks statistical support -- Pooled SEM cannot support claims of differences between Zhushan and Dabitou. Either conduct multi-group analysis or reframe as qualitative observations.

Response: As detailed in our response to Reviewer #1 (Comment 4), we have reframed all between-village contrasts as qualitative observations. We have added a clear statement in the Analytical Approach section explaining the decision not to perform MGA due to modest sub-sample sizes. The language in the Results section has been modified to anchor these contrasts in interview data and field notes.

Comment 4: Low factor loading for item P3 (0.53) -- This is below the conventional threshold of 0.60. Report model fit without P3 or justify its retention.

Response: We have addressed this with a sensitivity analysis (Page 15,L426). The analysis demonstrates that removing P3 results in only marginal changes to the path coefficient (β = 0.28 → 0.26, p remains significant) and model fit (R² = 0.63 → 0.62). Based on the robustness of the findings and the theoretical importance of "fair feedback" to the participation justice construct, we have justified its retention. This is a more transparent and rigorous approach than simply removing the item. The details of this analysis are presented in the Validity Analysis section.

Comment 5: Insufficient theoretical justification for rural-urban adaptation--- Explain why rural justice dynamics differ from urban (depopulation, tourism, aging) with references to rural geography literature.

Response: We have added a new, dedicated paragraph in the Introduction (Page 4, Line 157) titled "Why Rural Spatial Justice Requires Distinct Theoretical Treatment." This section systematically outlines three key structural differences between urban and rural contexts: demographic trajectories (depopulation/aging), political economy of spatial production (tourism/external capital), and the socio-cultural function of public space (collective memory/ritual). This addition provides a robust theoretical foundation for our rural-specific operationalization of spatial justice.

Comment 6: Missing chi-square p-value -- Report the p-value for the chi-square test (not just χ²/df = 2.31) and discuss implications for model fit.

Response: We thank the reviewer for this observation. However, following a comprehensive methodological revision, we have clarified that PLS-SEM is the sole analytical framework. As detailed in our response to Reviewer #1 (Comment 1), we have removed all covariance-based fit indices (including χ² and its associated p-value) from the manuscript. Reporting χ² is not appropriate or recommended for variance-based PLS-SEM. We have instead evaluated model quality using PLS-SEM specific criteria (SRMR, Q², and bootstrapped path significance), which we have reported in full.

Comment 7: Oral consent details insufficient -- Specify whether consent scripts were IRB-approved, how comprehension was verified, and how capacity was assessed for elderly participants.

Response: We have significantly expanded the ethics statement in the Questionnaire Design and Data Collection section (Page 11, Line325). The revised text now specifies that:The verbal script was reviewed and approved by the IRB as part of the protocol.Comprehension was verified by asking participants to restate key points in their own words.For elderly participants, interviewers were trained to assess alertness and orientation before proceeding.All consent outcomes were documented on field record sheets.

Comment 8: Data availability statement ambiguous -- Clarify whether raw survey data (314×16) will be published as supporting information or provide access conditions.

Response: We have added a clear Data Availability Statement after the Acknowledgments section (Page 32). The statement confirms that the minimal anonymized dataset has been uploaded as a Supporting Information file (S1 Dataset) , which will be made publicly available upon publication.

Comment 9: Social desirability bias not addressed -- Face-to-face interviews by researchers may bias responses about governance. Add a limitation paragraph and describe mitigation strategies.

Response: We have added a new subsection in the Limitations section (Page 30,Line 976 ) titled "Social Desirability Bias." This paragraph acknowledges the potential for bias in responses to governance-related questions, describes the mitigation strategies we employed (e.g., emphasizing anonymity, neutral phrasing, using local student interviewers), and notes that reported satisfaction levels may be positively biased. We also suggest methodological improvements for future research.

**Additional Revisions Made by the Authors**

Note on Figure 4 (Structural Equation Model Diagram): While the numerical data, path coefficients, and significance levels presented in Figure 4 remain unchangedfrom the original submission, we have replaced the figure file with a refined and more detailed version. The updated diagram provides a clearer and more precise visual representation of the measurement and structural model, improving readability for the audience. The source of the figure remains "Self-drawn by the author." This change is purely presentational and does not alter any of the reported results or scientific

content of the manuscript.

Corrected a citation error in the Results section: ‘Table 3-3’ has been changed to ‘Table 5’ (Page 21, Line 603)

**Responses to Journal Requirements (PLOS ONE)**

We have also addressed all points raised in the journal requirements email:

(1) Style Requirements: The manuscript has been reformatted according to the PLOS ONE Word template.
(2) Copy Editing: The revised manuscript has been carefully reviewed and edited by all co-authors to ensure clarity, grammatical accuracy, and adherence to standard academic English. While we did not use a formal third-party editing service, the manuscript has undergone multiple rounds of internal proofreading and language polishing.
(3) LaTeX Template: Not applicable. The manuscript was prepared in Microsoft Word.
(4) Funding Statement: We have removed all funding-related text from the Acknowledgments section. We request that the online Funding Statement be updated to: "This work was supported by the National Natural Science Foundation of China (grant number 42371254), the Scientific Research Project of Education Department of Hunan Province (grant number 23A0605), and the Scientific Research Project of Hunan Provincial Social Science Achievement Review Committee (grant number XSP2023YSC014). The funders had no role in study design, data collection and analysis, decision to publish, or preparation of the manuscript."
(5) Grant Numbers: We confirm that the grant numbers provided above are correct and match across all sections.
(6) Role of Funder: Included in the statement above.
(7) Data Availability: We confirm that the minimal data set has been uploaded as a Supporting Information file (S1_Dataset.xlsx).
(8) Figure/Table References: We have verified that all figures (Figures 1-5) and tables (Tables 1-12) are cited sequentially in the main text.
(9) Copyright for Figures 2 & 3: We have replaced the original map images. The new Figures 2 and 3 were created by the authors using Natural Earth public domain data. This is clearly stated in the figure captions.
(10) Copyright for Table 1: The photographs in Table 1 were taken by the authors during fieldwork. The caption has been updated to state: "(Source: Photo taken by the author.)"

We hope that these revisions and responses fully address the reviewers' and editor's concerns. We believe the manuscript is now significantly stronger and suitable for publication in PLOS ONE.

Thank you again for your valuable time and consideration.

Sincerely,

Bingbin Tian, Yunyuan Deng, and Mingcheng Jiang
