# Supplementary material for: Measurement of public space justice in traditional villages with coexistence of old and new based on villagers’ perceptions
Source: PLoS One. 2026 Jul 7;21(7):e0352578. doi: 10.1371/journal.pone.0352578 (PMC13340821; doi:10.1371/journal.pone.0352578)
Supplement: Attachment — Submitted filename: Reviwer Comments.docx [file pone.0352578.s003.docx]

**Comment:**

The authors have submitted a thoroughly revised manuscript that convincingly addresses the substantive methodological and theoretical concerns raised during the initial review. The revisions demonstrate a serious engagement with the feedback, significantly strengthening the paper's rigor, clarity, and scholarly contribution.

The key improvements that warrant acceptance are:

1. **Methodological Rigor and Transparency:** The authors have decisively clarified the analytical framework, explicitly stating the sole use of PLS-SEM and removing all inappropriate CB-SEM fit indices. The addition of a dedicated "Analytical Approach" subsection, the correction of terminology, and the inclusion of PLS-specific metrics (e.g., Q²) bring the methodology reporting to a high standard. The transparent presentation of the item-screening process (new Table 2) and the justification for retaining lower-loading indicators (e.g., P3) based on sensitivity analysis and theoretical grounds are commendable.
2. **Accurate and Consistent Reporting:** The critical inconsistency in the beta coefficient for distributive justice (Abstract vs. Results) has been corrected throughout the manuscript (β=0.45), ensuring internal consistency. All claims have been carefully tempered to align with the evidence.
3. **Appropriate Framing of Findings:** The authors have correctly reframed claims about "outperforming" one-dimensional models to more accurately highlight the value of their multidimensional approach in capturing interconnected perceptions. Crucially, they have reframed inter-village comparisons (Zhushan vs. Dabitou) as qualitative observations based on case evidence, adding a clear methodological statement that formal multi-group analysis was not conducted due to sample size. This is a responsible and appropriate interpretation of the data.
4. **Theoretical Strengthening:** The new paragraph in the Introduction, "Why Rural Spatial Justice Requires Distinct Theoretical Treatment," provides a much-needed and robust justification for adapting urban-centered justice theory to rural contexts, effectively addressing a core theoretical gap.
5. **Acknowledgment of Limitations and Refinement of Claims:** The authors have significantly expanded the Limitations section, thoughtfully addressing critical issues such as sample age bias, social desirability bias, and the illustrative (non-generalizable) nature of the proposed Spatial Justice Index (SJI). The language throughout the manuscript has been tempered, replacing overly causal claims with more measured interpretations.
6. **Ethical and Data Transparency:** The ethics statement has been expanded with sufficient detail on the oral consent procedure, and a clear Data Availability Statement has been added, meeting journal standards.

In summary, the revised manuscript is now methodologically sound, theoretically grounded, and clearly presented. The authors have successfully transformed a manuscript with potential into a robust and publishable research article that makes a valuable contribution to the fields of rural geography, spatial justice, and traditional village studies. The study's proposed three-dimensional model offers a practical, empirically validated tool for assessing justice perceptions in transitioning rural landscapes.

Therefore, I recommend **acceptance** of the manuscript in its current form.

**Decision:** **Accept**
