# Supplementary material for: Measurement of public space justice in traditional villages with coexistence of old and new based on villagers’ perceptions
Source: PLoS One. 2026 Jul 7;21(7):e0352578. doi: 10.1371/journal.pone.0352578 (PMC13340821; doi:10.1371/journal.pone.0352578)
Supplement: Peer review history

# Peer review: Decision Letter 0

**DOI:** 10.1371/journal.pone.0352578.r001

**Authors:** Jamil Afzal

6 Apr 2026

PONE-D-25-67060

Measurement of Public Space Justice in Traditional Villages with Coexistence of Old and New Based on Villagers’ Perceptions

PLOS One

Dear Dr. Deng,

Thank you for submitting your manuscript to PLOS ONE. After careful consideration, we feel that it has merit but does not fully meet PLOS ONE’s publication criteria as it currently stands. Therefore, we invite you to submit a revised version of the manuscript that addresses the points raised during the review process.

Please submit your revised manuscript by May 21 2026 11:59PM. If you will need more time than this to complete your revisions, please reply to this message or contact the journal office at plosone@plos.org. When you're ready to submit your revision, log on to https://www.editorialmanager.com/pone/ and select the 'Submissions Needing Revision' folder to locate your manuscript file.

Please include the following items when submitting your revised manuscript:

- A letter that responds to each point raised by the academic editor and reviewer(s). You should upload this letter as a separate file labeled 'Response to Reviewers'.
- A marked-up copy of your manuscript that highlights changes made to the original version. You should upload this as a separate file labeled 'Revised Manuscript with Track Changes'.
- An unmarked version of your revised paper without tracked changes. You should upload this as a separate file labeled 'Manuscript'.

If you would like to make changes to your financial disclosure, please include your updated statement in your cover letter. Guidelines for resubmitting your figure files are available below the reviewer comments at the end of this letter.

If applicable, we recommend that you deposit your laboratory protocols in protocols.io to enhance the reproducibility of your results. Protocols.io assigns your protocol its own identifier (DOI) so that it can be cited independently in the future. For instructions see: https://journals.plos.org/plosone/s/submission-guidelines#loc-laboratory-protocols. Additionally, PLOS ONE offers an option for publishing peer-reviewed Lab Protocol articles, which describe protocols hosted on protocols.io. Read more information on sharing protocols at https://plos.org/protocols?utm_medium=editorial-email&utm_source=authorletters&utm_campaign=protocols.

As the corresponding author, your ORCID iD is verified in the submission system and will appear in the published article. PLOS supports the use of ORCID, and we encourage all coauthors to register for an ORCID iD and use it as well. Please encourage your coauthors to verify their ORCID iD within the submission system before final acceptance, as unverified ORCID iDs will not appear in the published article. *Only*  the individual author can complete the verification step; PLOS staff *cannot*  verify ORCID iDs on behalf of authors.

We look forward to receiving your revised manuscript.

Kind regards,

Jamil Afzal, Ph.D, Post Doc

Academic Editor

PLOS One

Journal Requirements:

When submitting your revision, we need you to address these additional requirements.

1. Please ensure that your manuscript meets PLOS ONE's style requirements, including those for file naming. The PLOS ONE style templates can be found at

https://journals.plos.org/plosone/s/file?id=wjVg/PLOSOne_formatting_sample_main_body.pdf and

https://journals.plos.org/plosone/s/file?id=ba62/PLOSOne_formatting_sample_title_authors_affiliations.pdf

2. Please request copy editing

3. Please update your submission to use the PLOS LaTeX template. The template and more information on our requirements for LaTeX submissions can be found at http://journals.plos.org/plosone/s/latex.

4. Thank you for stating the following in the Acknowledgments Section of your manuscript:

“I would like to express my sincere gratitude to my supervisor, Professor Deng Yunyuan, for his patient guidance and invaluable support. Thanks to his assistance, the overall structure and innovative aspects of this thesis have become much clearer. He also provided financial support during my multiple field trips to traditional villages, such as Zhushan Village and Dabidou Village.”

We note that you have provided funding information that is not currently declared in your Funding Statement. However, funding information should not appear in the Acknowledgments section or other areas of your manuscript. We will only publish funding information present in the Funding Statement section of the online submission form.

Please remove any funding-related text from the manuscript and let us know how you would like to update your Funding Statement. Currently, your Funding Statement reads as follows:

“This work was supported by National Natural Science Foundation of China (42371254), and Scientific Research Project of Education Department of Hunan Province (23A0605) and Scientific Research Project of Hunan Provincial Social Science Achievement Review Committee (XSP2023YSC014).”

Please include your amended statements within your cover letter; we will change the online submission form on your behalf.

5. We note that the grant information you provided in the ‘Funding Information’ and ‘Financial Disclosure’ sections do not match.

When you resubmit, please ensure that you provide the correct grant numbers for the awards you received for your study in the ‘Funding Information’ section.

6. Thank you for stating the following financial disclosure:

“This work was supported by National Natural Science Foundation of China (42371254), and Scientific Research Project of Education Department of Hunan Province (23A0605) and Scientific Research Project of Hunan Provincial Social Science Achievement Review Committee (XSP2023YSC014).”

Please state what role the funders took in the study.  If the funders had no role, please state: "The funders had no role in study design, data collection and analysis, decision to publish, or preparation of the manuscript."

If this statement is not correct you must amend it as needed.

Please include this amended Role of Funder statement in your cover letter; we will change the online submission form on your behalf.

7. We note that your Data Availability Statement is currently as follows: “All relevant data are within the manuscript and its Supporting Information files.”

Please confirm at this time whether or not your submission contains all raw data required to replicate the results of your study. Authors must share the “minimal data set” for their submission. PLOS defines the minimal data set to consist of the data required to replicate all study findings reported in the article, as well as related metadata and methods (https://journals.plos.org/plosone/s/data-availability#loc-minimal-data-set-definition).

For example, authors should submit the following data:

- The values behind the means, standard deviations and other measures reported;

- The values used to build graphs;

- The points extracted from images for analysis.

Authors do not need to submit their entire data set if only a portion of the data was used in the reported study.

If your submission does not contain these data, please either upload them as Supporting Information files or deposit them to a stable, public repository and provide us with the relevant URLs, DOIs, or accession numbers. For a list of recommended repositories, please see https://journals.plos.org/plosone/s/recommended-repositories.

If there are ethical or legal restrictions on sharing a de-identified data set, please explain them in detail (e.g., data contain potentially sensitive information, data are owned by a third-party organization, etc.) and who has imposed them (e.g., an ethics committee). Please also provide contact information for a data access committee, ethics committee, or other institutional body to which data requests may be sent. If data are owned by a third party, please indicate how others may request data access.

8. Please ensure that you refer to Figures 1 - 5 in your text as, if accepted, production will need this reference to link the reader to the figure.

9. We note that Figures 2 and 3 in your submission contain map images which may be copyrighted. All PLOS content is published under the Creative Commons Attribution License (CC BY 4.0), which means that the manuscript, images, and Supporting Information files will be freely available online, and any third party is permitted to access, download, copy, distribute, and use these materials in any way, even commercially, with proper attribution. For these reasons, we cannot publish previously copyrighted maps or satellite images created using proprietary data, such as Google software (Google Maps, Street View, and Earth). For more information, see our copyright guidelines: http://journals.plos.org/plosone/s/licenses-and-copyright.

We require you to either (1) present written permission from the copyright holder to publish these figures specifically under the CC BY 4.0 license, or (2) remove the figures from your submission:

1. You may seek permission from the original copyright holder of Figure(s) [#] to publish the content specifically under the CC BY 4.0 license.

We recommend that you contact the original copyright holder with the Content Permission Form (http://journals.plos.org/plosone/s/file?id=7c09/content-permission-form.pdf) and the following text:

“I request permission for the open-access journal PLOS ONE to publish XXX under the Creative Commons Attribution License (CCAL) CC BY 4.0 (http://creativecommons.org/licenses/by/4.0/). Please be aware that this license allows unrestricted use and distribution, even commercially, by third parties. Please reply and provide explicit written permission to publish XXX under a CC BY license and complete the attached form.”

Please upload the completed Content Permission Form or other proof of granted permissions as an "Other" file with your submission.

In the figure caption of the copyrighted figure, please include the following text: “Reprinted from [ref] under a CC BY license, with permission from [name of publisher], original copyright [original copyright year].”

2. If you are unable to obtain permission from the original copyright holder to publish these figures under the CC BY 4.0 license or if the copyright holder’s requirements are incompatible with the CC BY 4.0 license, please either i) remove the figure or ii) supply a replacement figure that complies with the CC BY 4.0 license. Please check copyright information on all replacement figures and update the figure caption with source information. If applicable, please specify in the figure caption text when a figure is similar but not identical to the original image and is therefore for illustrative purposes only.

The following resources for replacing copyrighted map figures may be helpful:

USGS National Map Viewer (public domain): http://viewer.nationalmap.gov/viewer/

The Gateway to Astronaut Photography of Earth (public domain): http://eol.jsc.nasa.gov/sseop/clickmap/

Maps at the CIA (public domain): https://www.cia.gov/library/publications/the-world-factbook/index.html and https://www.cia.gov/library/publications/cia-maps-publications/index.html

NASA Earth Observatory (public domain): http://earthobservatory.nasa.gov/

Landsat: http://landsat.visibleearth.nasa.gov/

USGS EROS (Earth Resources Observatory and Science (EROS) Center) (public domain): http://eros.usgs.gov/#

Natural Earth (public domain): http://www.naturalearthdata.com/

10. We note you have included a table to which you do not refer in the text of your manuscript. Please ensure that you refer to Tables 1 - 6 and 12 in your text; if accepted, production will need this reference to link the reader to the Table.

11. We note that Table 1 in your submission contain copyrighted images. All PLOS content is published under the Creative Commons Attribution License (CC BY 4.0), which means that the manuscript, images, and Supporting Information files will be freely available online, and any third party is permitted to access, download, copy, distribute, and use these materials in any way, even commercially, with proper attribution. For more information, see our copyright guidelines: http://journals.plos.org/plosone/s/licenses-and-copyright.

We require you to either (1) present written permission from the copyright holder to publish these figures specifically under the CC BY 4.0 license, or (2) remove the figures from your submission:

1. You may seek permission from the original copyright holder of Figure(s) [#] to publish the content specifically under the CC BY 4.0 license.

We recommend that you contact the original copyright holder with the Content Permission Form (http://journals.plos.org/plosone/s/file?id=7c09/content-permission-form.pdf) and the following text:

“I request permission for the open-access journal PLOS ONE to publish XXX under the Creative Commons Attribution License (CCAL) CC BY 4.0 (http://creativecommons.org/licenses/by/4.0/). Please be aware that this license allows unrestricted use and distribution, even commercially, by third parties. Please reply and provide explicit written permission to publish XXX under a CC BY license and complete the attached form.”

Please upload the completed Content Permission Form or other proof of granted permissions as an "Other" file with your submission.

In the figure caption of the copyrighted figure, please include the following text: “Reprinted from [ref] under a CC BY license, with permission from [name of publisher], original copyright [original copyright year].”

2. If you are unable to obtain permission from the original copyright holder to publish these figures under the CC BY 4.0 license or if the copyright holder’s requirements are incompatible with the CC BY 4.0 license, please either i) remove the figure or ii) supply a replacement figure that complies with the CC BY 4.0 license. Please check copyright information on all replacement figures and update the figure caption with source information. If applicable, please specify in the figure caption text when a figure is similar but not identical to the original image and is therefore for illustrative purposes only.

12. If the reviewer comments include a recommendation to cite specific previously published works, please review and evaluate these publications to determine whether they are relevant and should be cited. There is no requirement to cite these works unless the editor has indicated otherwise.

[Note: HTML markup is below. Please do not edit.]

Reviewers' comments:

Reviewer's Responses to Questions

**Comments to the Author**

1. Is the manuscript technically sound, and do the data support the conclusions?

The manuscript must describe a technically sound piece of scientific research with data that supports the conclusions. Experiments must have been conducted rigorously, with appropriate controls, replication, and sample sizes. The conclusions must be drawn appropriately based on the data presented.

Reviewer #1: Partly

Reviewer #2: Partly

**********

2. Has the statistical analysis been performed appropriately and rigorously?

Reviewer #1: No

Reviewer #2: Yes

**********

3. Have the authors made all data underlying the findings in their manuscript fully available?

The PLOS Data policy requires authors to make all data underlying the findings described in their manuscript fully available without restriction, with rare exception (please refer to the Data Availability Statement in the manuscript PDF file). The data should be provided as part of the manuscript or its supporting information, or deposited to a public repository. For example, in addition to summary statistics, the data points behind means, medians and variance measures should be available. If there are restrictions on publicly sharing data—e.g. participant privacy or use of data from a third party—those must be specified.

Reviewer #1: No

Reviewer #2: Yes

**********

4. Is the manuscript presented in an intelligible fashion and written in standard English?

PLOS ONE does not copyedit accepted manuscripts, so the language in submitted articles must be clear, correct, and unambiguous. Any typographical or grammatical errors should be corrected at revision, so please note any specific errors here.

Reviewer #1: Yes

Reviewer #2: No

**********

5. Review Comments to the Author

Please use the space provided to explain your answers to the questions above. You may also include additional comments for the author, including concerns about dual publication, research ethics, or publication ethics. (Please upload your review as an attachment if it exceeds 20,000 characters)

Reviewer #1: The manuscript is partly technically sound. It presents a clearly described two-site field survey (314 valid responses collected through face-to-face interviews in Zhushan in October 2024 and Dabitou in April 2025) and reports a standard set of measurement checks, including reliability and validity indicators (e.g., Cronbach’s alpha, KMO/Bartlett, composite reliability/AVE, and factor loadings), alongside structural path estimation using bootstrapping in SmartPLS (1,000 subsamples). Nevertheless, several internal inconsistencies and gaps in evidence weaken confidence that the conclusions are fully supported by the reported analyses. In particular, the manuscript mixes and conflates reporting conventions from PLS-SEM and CB-SEM, contains a mismatch between abstracted and tabulated coefficients, and advances comparative claims (e.g., “outperforming” one-dimensional models and “spatial differences” between villages) without clearly presenting the required baseline models, statistical comparisons, or formal tests.

First, the statistical methodology is not reported consistently. While the manuscript describes the use of SmartPLS and the PLS algorithm with bootstrapping, it simultaneously reports CB-SEM-style global fit indices such as χ²/df, CFI, TLI, and RMSEA. This blending of paradigms is confusing and makes it difficult to assess technical adequacy. The authors should explicitly state whether the primary analytical framework is PLS-SEM or CB-SEM and report diagnostics appropriate to that choice. If both approaches were genuinely used (e.g., AMOS alongside PLS, as suggested in the acknowledgments), the manuscript must clearly justify the dual approach, specify which software was used at each stage (measurement versus structural model estimation), and present results in a coherent, methodologically aligned manner.

Second, there is a key inconsistency between the abstract and the results section. The abstract reports the effect of distributive justice as β = 0.58, whereas the structural model table reports β = 0.45 for Distribution Justice predicting overall justice perception. This discrepancy should be corrected throughout the manuscript so that all reported coefficients match the final model output.

Third, the claim that the proposed framework “outperforms traditional one-dimensional models” is not currently demonstrated. Although the model is reported to explain 63% of the variance (R² = 0.63), the manuscript does not provide the results of any one-dimensional baseline models (e.g., Recognition-only, Distribution-only, Participation-only), nor does it report an explicit model comparison using a consistent criterion (e.g., ΔR², predictive performance, or other appropriate SEM comparison metrics). Without such baseline estimation and comparison, the conclusion of “outperformance” is not supported by the evidence presented.

Fourth, the manuscript’s claims of “important spatial differences” between villages are not adequately substantiated with explicit quantitative testing. The abstract advances mechanism-level explanations (e.g., capital-driven monopolies suppress participation in Dabitou, while governance structures strengthen recognition in Zhushan), and the Results/Discussion provides narrative “case evidence,” yet it remains unclear what statistical tests support between-village differences. If the authors intend this as a quantitative comparison, they should report per-village sample sizes and conduct appropriate statistical analyses (e.g., mean comparisons on constructs, multi-group analysis in SEM, and—crucially—measurement invariance testing where relevant). If the inference is primarily qualitative, then the paper should describe the qualitative design, sampling, and analytic procedures more rigorously and temper causal language accordingly.

Fifth, measurement development is insufficiently transparent and contains contradictions. The manuscript states that 25 items were reduced by removing 9 low-loading items, leaving 16 items; however, it later refers to “removing two items” while still retaining “the remaining 16 items,” which is mathematically inconsistent unless intermediate versions existed. In addition, several retained indicators have notably low factor loadings (e.g., P3 = 0.53 and distribution indicators as low as ~0.61), despite the earlier claim that low-loading items had already been eliminated. The authors should provide a clear item-retention flow (initial pool → pretest modifications → final instrument), explicitly list removed items at each step, justify the retention of lower-loading indicators (or revise/remove them), and demonstrate whether conclusions are robust to alternative measurement specifications.

Sixth, the proposed Spatial Justice Index (SJI) weighting scheme appears potentially circular and requires stronger justification. The index is presented as SJI = 0.45×Distributive + 0.32×Recognition + 0.23×Participation, apparently derived from standardized model effects. This risks overfitting because the weights are extracted from the same sample/model used to validate the index, yet the index is framed as an operational tool. The authors should strengthen the rationale for the weights (normative and/or empirical) and include sensitivity analyses (e.g., equal weights, alternative weighting schemes, or cross-validation). If model-derived weights are retained, the index should be presented as context- and sample-specific rather than generalized.

Finally, several additional points should be addressed to strengthen rigor and presentation. The manuscript reports bootstrapping with 1,000 subsamples; this is often considered minimal in SEM practice, so increasing the number of resamples and reporting confidence intervals for path coefficients would improve stability and interpretability. Moreover, some proposed policy thresholds (e.g., “30% attendance” or “50% agenda items co-proposed”) read as normative and are not clearly derived from the presented data, so these should be justified empirically or softened in tone. The limitations (e.g., underrepresentation of youth and reliance on perceptual survey data) are appropriately acknowledged, but the abstract and discussion should reflect these limitations more consistently by avoiding overly strong causal language and by framing findings as associative rather than deterministic where warranted.

Reviewer #2: 1. Spatial Justice Index (SJI) derivation is statistically invalid – Path coefficients cannot be directly used as policy weights. Remove the SJI formula or clearly state it is illustrative only, with proper justification.

2. Severe age bias undermines generalizability – Only 9.6% of respondents are under 25. Temper claims about global scalability and frame findings as specific to aging, low-education rural populations.

3. Village comparison lacks statistical support – Pooled SEM cannot support claims of differences between Zhushan and Dabitou. Either conduct multi-group analysis or reframe as qualitative observations.

4. Low factor loading for item P3 (0.53) – This is below the conventional threshold of 0.60. Report model fit without P3 or justify its retention.

5. Insufficient theoretical justification for rural-urban adaptation— Explain why rural justice dynamics differ from urban (depopulation, tourism, aging) with references to rural geography literature.

6. Missing chi-square p-value – Report the p-value for the chi-square test (not just χ²/df = 2.31) and discuss implications for model fit.

7. Oral consent details insufficient – Specify whether consent scripts were IRB-approved, how comprehension was verified, and how capacity was assessed for elderly participants.

8. Data availability statement ambiguous – Clarify whether raw survey data (314×16) will be published as supporting information or provide access conditions.

9. Social desirability bias not addressed – Face-to-face interviews by researchers may bias responses about governance. Add a limitation paragraph and describe mitigation strategies.

**********

6. PLOS authors have the option to publish the peer review history of their article (what does this mean?). If published, this will include your full peer review and any attached files.

If you choose “no”, your identity will remain anonymous but your review may still be made public.

**Do you want your identity to be public for this peer review?**  For information about this choice, including consent withdrawal, please see our Privacy Policy.

Reviewer #1: **Yes:** MOHD NORBAYUSRI BAHARUDIN

Reviewer #2: No

**********

[NOTE: If reviewer comments were submitted as an attachment file, they will be attached to this email and accessible via the submission site. Please log into your account, locate the manuscript record, and check for the action link "View Attachments". If this link does not appear, there are no attachment files.]

To ensure your figures meet our technical requirements, please review our figure guidelines: https://journals.plos.org/plosone/s/figures

You may also use PLOS’s free figure tool, NAAS, to help you prepare publication quality figures: https://journals.plos.org/plosone/s/figures#loc-tools-for-figure-preparation.

NAAS will assess whether your figures meet our technical requirements by comparing each figure against our figure specifications.

# Author comment: Author response to Decision Letter 1

**DOI:** 10.1371/journal.pone.0352578.r002

27 Apr 2026

Responses to Reviewer #1

Reviewer's Summary: The manuscript is partly technically sound... several internal inconsistencies and gaps in evidence weaken confidence that the conclusions are fully supported by the reported analyses.

Response: We thank the reviewer for this thorough and constructive assessment. We have carefully addressed each of the specific points raised, as detailed below.

Comment 1: The statistical methodology is not reported consistently. While the manuscript describes the use of SmartPLS and the PLS algorithm with bootstrapping, it simultaneously reports CB-SEM-style global fit indices... The authors should explicitly state whether the primary analytical framework is PLS-SEM or CB-SEM and report diagnostics appropriate to that choice.

Response: We fully acknowledge this critical inconsistency in the original submission. We have taken the following steps to rectify this issue:

(1)Clarified Analytical Framework: We have added a dedicated subsection titled "Analytical Approach" in the Materials and methods section (Page12, Line 359-368). This section explicitly states that Partial Least Squares Structural Equation Modeling (PLS-SEM) was the sole analytical framework employed, justified by the study's prediction-oriented nature and moderate sample size. We confirm that all analyses were conducted using Smart PLS 3.0.

(2)Removed Inappropriate Fit Indices: We have completely deleted the former Table 11 (Structural Model Fit Indices) and all associated text that reported CB-SEM metrics such as χ²/df, CFI, TLI, and RMSEA. These indices are not appropriate for variance-based PLS-SEM.

(3)Corrected Terminology: In the Path Analysis section and in Table 12, we have replaced the CB-SEM term "C.R. Value" with the PLS-SEM standard "T Statistics".

(4)Added PLS-SEM Specific Metrics: We have supplemented the model evaluation with the Stone-Geisser's Q² value to assess predictive relevance. As reported in the Variance Explained and Effect Size Test section (Page 18, Line 525), the blindfolding procedure yielded a Q² value of 0.625, confirming the model's predictive validity.

(5)Corrected Acknowledgments: We have removed the reference to AMOS from the Acknowledgments section to ensure consistency with the sole use of Smart PLS.

Comment 2: There is a key inconsistency between the abstract and the results section. The abstract reports the effect of distributive justice as β = 0.58, whereas the structural model table reports β = 0.45...

Response: We apologize for this typographical error. The correct standardized path coefficient is β = 0.45. We have corrected this throughout the entire manuscript, including the Abstract (Page 1, Line15), to ensure consistency with the final model output presented in Table 12.

Comment 3: The claim that the proposed framework "outperforms traditional one-dimensional models" is not currently demonstrated...

Response: We agree with the reviewer that the original phrasing was not supported by a formal model comparison. Following the reviewer's guidance, we have removed all claims of "outperforming" one-dimensional models. In the revised Abstract (Page 1, Line 10]), the text now emphasizes the value of the multidimensional framework in capturing the interconnected nature of justice perceptions, stating: "...highlighting the interconnected nature of recognition, distribution, and participation in shaping justice perceptions, a nuance that single-dimension approaches may overlook." We believe this more accurately reflects the contribution of our study without overstating the empirical evidence.

Comment 4: The manuscript's claims of "important spatial differences" between villages are not adequately substantiated with explicit quantitative testing... Either conduct multi-group analysis or reframe as qualitative observations.

Response: This is an excellent point. We have clarified that our discussion of village-specific dynamics is based on qualitative case evidence rather than statistical multi-group analysis. The following revisions were made:

(1) Methodological Clarification: In the Analytical Approach section (Page 12, Line 369-374), we have added a statement: "A total of 314 valid responses were collected, comprising 149 from Zhushan Village and 165 from Dabitou Village. Given the modest sub-sample sizes, formal multi-group analysis was not conducted; village-level comparisons in this study are therefore based on qualitative case evidence rather than statistical inference."

(2)Tempered Language: We have revised the language throughout the Abstract and Results sections to clearly frame spatial contrasts as qualitative observations. For example, the Abstract now states: "Qualitative contrasts suggest how endogenous governance in Zhushan may strengthen recognition, while capital-driven monopolies in Dabitou appear to suppress participation." In the Results section (Page 1, Line 17-20), we anchor these observations in interview excerpts and explicitly note that they are illustrative case contrasts.

Comment 5: Measurement development is insufficiently transparent and contains contradictions... provide a clear item-retention flow... justify the retention of lower-loading indicators.

Response: We have thoroughly revised this section for transparency and accuracy.

①　Item-Retention Flow: We have added a new Table 2 (Item Screening and Retention Process) in the Questionnaire Design section (Page 10), which clearly outlines the three stages from the initial 25-item pool to the final 16-item instrument.

②　Contradiction Removed: The contradictory sentence "After removing two items..." has been removed. The text now clearly states that 9 items were removed during the pre-test, resulting in the final 16 items.

③　Justification for Lower Loadings: We thank the reviewer for this important methodological observation. We have addressed this concern through both empirical sensitivity analysis and transparent theoretical justification, as detailed below:1.Sensitivity Analysis. We re-estimated the structural model after temporarily removing P3 from the participation justice construct. The key results are summarized in the table below (reproduced from our response to Reviewer #1, Comment 5, for ease of reference):The analysis demonstrates that removing P3 results in only marginal changes to the path coefficient (β = 0.28 → 0.26, p remains <0.01) and model fit (R² = 0.63 → 0.62). The substantive conclusions of the study are unchanged.

指标 保留P3的原始模型 删除P3后的模型 变化

Participation Justice的CR 0.82 0.85 +0.03

Participation Justice的AVE 0.53 0.58 +0.05

路系数�Participation → Overall Perception 0.28 0.26 -0.02

该路径的T Statistics 4.67 4.21 仍显著(p<0.001)

该路径的P Value 0.003 0.005 仍<0.01

Overall Perception的R² 0.63 0.62 -0.01

Participation Justice的f2 0.08 0.07 -0.01

④　Justification for Retention. Based on the robustness of the findings and the theoretical importance of “fair feedback” to the participation justice construct, we elected to retain P3. This decision follows established PLS-SEM guidelines indicating that loadings between 0.40 and 0.70 are acceptable in exploratory social science research when the indicator contributes substantively to the construct’s theoretical domain and its removal does not compromise composite reliability or average variance extracted.

⑤　Acknowledgment of Limitation and Path Forward. We acknowledge that the loading of P3 (0.53) remains below the conventional threshold of 0.60. While our sensitivity analysis confirms that its removal does not alter the substantive conclusions, future scale development efforts should refine or replace this item (e.g., by rewording “fair feedback” more concretely or splitting it into multiple items) to improve psychometric robustness. We have noted this limitation in the revised manuscript (Validity Analysis section, Page 15, Line 426-435) and again in the Limitations section (Page 31, Line 998-1002) to guide future research.

Comment 6: The proposed Spatial Justice Index (SJI) weighting scheme appears potentially circular... should be presented as context- and sample-specific rather than generalized.

Response: We fully concur with this insightful methodological critique. We have significantly revised the presentation of the SJI in the Limitations and Future Research Directions section (Page 30, Line 888). The formula is now described as an "exploratory and illustrative formulation" and a "context-specific, sample-dependent illustration." We have added strong caveats stating: "We emphasize that this formulation is statistically derived from a single sample and should not be interpreted as a generalizable policy weight... This index is presented solely to illustrate how empirical findings might inform the conceptual development of future assessment tools, pending rigorous cross-validation and sensitivity testing." We have also removed language that framed it as an "operational tool."

Comment 7: Several additional points... bootstrapping with 1,000 subsamples... policy thresholds... limitations...

Response: We have addressed each of these points as follows:

(1)Bootstrapping: We have re-run the analysis with 5,000 subsamples (previously 1,000). The updated T Statistics and P Values are reported in Table 12 and referenced in the Path Analysis section (Page19,Line 547).

(2)Policy Thresholds: We have removed the specific, unsupported numeric thresholds (e.g., "30% attendance") from the Limitations section. The text now speaks in terms of general principles and benchmarks that would require further empirical calibration.

(3)Limitations and Language: We have significantly expanded the Limitations section (Page30, Line 939) to explicitly discuss the age bias in the sample and the potential for social desirability bias. We have also performed a full pass of the manuscript to temper causal language, replacing terms like "demonstrates" with "suggests" or "indicates" where appropriate.

Responses to Reviewer #2

Comment 1: Spatial Justice Index (SJI) derivation is statistically invalid -- Path coefficients cannot be directly used as policy weights. Remove the SJI formula or clearly state it is illustrative only, with proper justification.

Response: As noted in our response to Reviewer #1 (Comment 6), we have thoroughly revised the SJI section. We have retained the formula solely for illustrative purposes and have added explicit, strong caveats that it is not a validated policy weight and is specific to this sample. We hope this addresses the reviewer's statistical validity concern.

Comment 2: Severe age bias undermines generalizability -- Only 9.6% of respondents are under 25. Temper claims about global scalability and frame findings as specific to aging, low-education rural populations.

Response: We agree. We have taken the following actions:

(1)Revised Abstract and Conclusion: We have removed claims of global scalability. The Abstract (Page1,L22) now describes the metric as "preliminary" and relevant for "similar aging rural contexts."

(2)Expanded Limitations: We have added a dedicated subsection titled "Sample Representativeness and Age Bias" in the Limitations section (Page30,L939). This section details the age distribution, discusses the underrepresentation of youth, and explicitly cautions against generalizing the findings to younger or demographically different populations.

(3)Tempered language in the Author summary: We have further tempered the language in the Author summary by adding an explicit applicability caveat. Specifically, we now state that the proposed tool “is most directly applicable to aging, depopulating rural contexts and should be validated in younger populations before broader generalization.” This change ensures that the summary accurately reflects the sample limitations and avoids overstatement of generalizability.

Comment 3: Village comparison lacks statistical support -- Pooled SEM cannot support claims of differences between Zhushan and Dabitou. Either conduct multi-group analysis or reframe as qualitative observations.

Response: As detailed in our response to Reviewer #1 (Comment 4), we have reframed all between-village contrasts as qualitative observations. We have added a clear statement in the Analytical Approach section explaining the decision not to perform MGA due to modest sub-sample sizes. The language in the Results section has been modified to anchor these contrasts in interview data and field notes.

Comment 4: Low factor loading for item P3 (0.53) -- This is below the conventional threshold of 0.60. Report model fit without P3 or justify its retention.

Response: We have addressed this with a sensitivity analysis (Page 15,L426). The analysis demonstrates that removing P3 results in only marginal changes to the path coefficient (β = 0.28 → 0.26, p remains significant) and model fit (R² = 0.63 → 0.62). Based on the robustness of the findings and the theoretical importance of "fair feedback" to the participation justice construct, we have justified its retention. This is a more transparent and rigorous approach than simply removing the item. The details of this analysis are presented in the Validity Analysis section.

Comment 5: Insufficient theoretical justification for rural-urban adaptation--- Explain why rural justice dynamics differ from urban (depopulation, tourism, aging) with references to rural geography literature.

Response: We have added a new, dedicated paragraph in the Introduction (Page 4, Line 157) titled "Why Rural Spatial Justice Requires Distinct Theoretical Treatment." This section systematically outlines three key structural differences between urban and rural contexts: demographic trajectories (depopulation/aging), political economy of spatial production (tourism/external capital), and the socio-cultural function of public space (collective memory/ritual). This addition provides a robust theoretical foundation for our rural-specific operationalization of spatial justice.

Comment 6: Missing chi-square p-value -- Report the p-value for the chi-square test (not just χ²/df = 2.31) and discuss implications for model fit.

Response: We thank the reviewer for this observation. However, following a comprehensive methodological revision, we have clarified that PLS-SEM is the sole analytical framework. As detailed in our response to Reviewer #1 (Comment 1), we have removed all covariance-based fit indices (including χ² and its associated p-value) from the manuscript. Reporting χ² is not appropriate or recommended for variance-based PLS-SEM. We have instead evaluated model quality using PLS-SEM specific criteria (SRMR, Q², and bootstrapped path significance), which we have reported in full.

Comment 7: Oral consent details insufficient -- Specify whether consent scripts were IRB-approved, how comprehension was verified, and how capacity was assessed for elderly participants.

Response: We have significantly expanded the ethics statement in the Questionnaire Design and Data Collection section (Page 11, Line325). The revised text now specifies that:The verbal script was reviewed and approved by the IRB as part of the protocol.Comprehension was verified by asking participants to restate key points in their own words.For elderly participants, interviewers were trained to assess alertness and orientation before proceeding.All consent outcomes were documented on field record sheets.

Comment 8: Data availability statement ambiguous -- Clarify whether raw survey data (314×16) will be published as supporting information or provide access conditions.

Response: We have added a clear Data Availability Statement after the Acknowledgments section (Page 32). The statement confirms that the minimal anonymized dataset has been uploaded as a Supporting Information file (S1 Dataset) , which will be made publicly available upon publication.

Comment 9: Social desirability bias not addressed -- Face-to-face interviews by researchers may bias responses about governance. Add a limitation paragraph and describe mitigation strategies.

Response: We have added a new subsection in the Limitations section (Page 30,Line 976 ) titled "Social Desirability Bias." This paragraph acknowledges the potential for bias in responses to governance-related questions, describes the mitigation strategies we employed (e.g.,

Attachment Submitted filename: Response to Reviewers.docx

# Peer review: Decision Letter 1

**DOI:** 10.1371/journal.pone.0352578.r003

**Authors:** Jamil Afzal

11 Jun 2026

Measurement of Public Space Justice in Traditional Villages with Coexistence of Old and New Based on Villagers' Perceptions

PONE-D-25-67060R1

Dear Dr. Deng,

We’re pleased to inform you that your manuscript has been judged scientifically suitable for publication and will be formally accepted for publication once it meets all outstanding technical requirements.

Within one week, you’ll receive an e-mail detailing the required amendments. When these have been addressed, you’ll receive a formal acceptance letter and your manuscript will be scheduled for publication.

An invoice will be generated when your article is formally accepted. Please note, if your institution has a publishing partnership with PLOS and your article meets the relevant criteria, all or part of your publication costs will be covered. Please make sure your user information is up-to-date by logging into Editorial Manager at Editorial Manager® and clicking the ‘Update My Information' link at the top of the page. For questions related to billing, please contact billing support.

If your institution or institutions have a press office, please notify them about your upcoming paper to help maximize its impact. If they’ll be preparing press materials, please inform our press team as soon as possible -- no later than 48 hours after receiving the formal acceptance. Your manuscript will remain under strict press embargo until 2 pm Eastern Time on the date of publication. For more information, please contact onepress@plos.org.

Kind regards,

Jamil Afzal, Ph.D, Post Doc

Academic Editor

PLOS One

Additional Editor Comments (optional):

Reviewers' comments:

Reviewer's Responses to Questions

**Comments to the Author**

1. If the authors have adequately addressed your comments raised in a previous round of review and you feel that this manuscript is now acceptable for publication, you may indicate that here to bypass the “Comments to the Author” section, enter your conflict of interest statement in the “Confidential to Editor” section, and submit your "Accept" recommendation.

Reviewer #2: All comments have been addressed

Reviewer #3: All comments have been addressed

**********

2. Is the manuscript technically sound, and do the data support the conclusions?

The manuscript must describe a technically sound piece of scientific research with data that supports the conclusions. Experiments must have been conducted rigorously, with appropriate controls, replication, and sample sizes. The conclusions must be drawn appropriately based on the data presented.

Reviewer #2: Yes

Reviewer #3: Yes

**********

3. Has the statistical analysis been performed appropriately and rigorously?

Reviewer #2: Yes

Reviewer #3: Yes

**********

4. Have the authors made all data underlying the findings in their manuscript fully available?

The PLOS Data policy requires authors to make all data underlying the findings described in their manuscript fully available without restriction, with rare exception (please refer to the Data Availability Statement in the manuscript PDF file). The data should be provided as part of the manuscript or its supporting information, or deposited to a public repository. For example, in addition to summary statistics, the data points behind means, medians and variance measures should be available. If there are restrictions on publicly sharing data—e.g. participant privacy or use of data from a third party—those must be specified.

Reviewer #2: Yes

Reviewer #3: Yes

**********

5. Is the manuscript presented in an intelligible fashion and written in standard English?

PLOS ONE does not copyedit accepted manuscripts, so the language in submitted articles must be clear, correct, and unambiguous. Any typographical or grammatical errors should be corrected at revision, so please note any specific errors here.

Reviewer #2: Yes

Reviewer #3: Yes

**********

6. Review Comments to the Author

Please use the space provided to explain your answers to the questions above. You may also include additional comments for the author, including concerns about dual publication, research ethics, or publication ethics. (Please upload your review as an attachment if it exceeds 20,000 characters)

Reviewer #2: (No Response)

Reviewer #3: Comment:

The authors have submitted a thoroughly revised manuscript that convincingly addresses the substantive methodological and theoretical concerns raised during the initial review. The revisions demonstrate a serious engagement with the feedback, significantly strengthening the paper's rigor, clarity, and scholarly contribution.

The key improvements that warrant acceptance are:

1. Methodological Rigor and Transparency: The authors have decisively clarified the analytical framework, explicitly stating the sole use of PLS-SEM and removing all inappropriate CB-SEM fit indices. The addition of a dedicated "Analytical Approach" subsection, the correction of terminology, and the inclusion of PLS-specific metrics (e.g., Q²) bring the methodology reporting to a high standard. The transparent presentation of the item-screening process (new Table 2) and the justification for retaining lower-loading indicators (e.g., P3) based on sensitivity analysis and theoretical grounds are commendable.

2. Accurate and Consistent Reporting: The critical inconsistency in the beta coefficient for distributive justice (Abstract vs. Results) has been corrected throughout the manuscript (β=0.45), ensuring internal consistency. All claims have been carefully tempered to align with the evidence.

3. Appropriate Framing of Findings: The authors have correctly reframed claims about "outperforming" one-dimensional models to more accurately highlight the value of their multidimensional approach in capturing interconnected perceptions. Crucially, they have reframed inter-village comparisons (Zhushan vs. Dabitou) as qualitative observations based on case evidence, adding a clear methodological statement that formal multi-group analysis was not conducted due to sample size. This is a responsible and appropriate interpretation of the data.

4. Theoretical Strengthening: The new paragraph in the Introduction, "Why Rural Spatial Justice Requires Distinct Theoretical Treatment," provides a much-needed and robust justification for adapting urban-centered justice theory to rural contexts, effectively addressing a core theoretical gap.

5. Acknowledgment of Limitations and Refinement of Claims: The authors have significantly expanded the Limitations section, thoughtfully addressing critical issues such as sample age bias, social desirability bias, and the illustrative (non-generalizable) nature of the proposed Spatial Justice Index (SJI). The language throughout the manuscript has been tempered, replacing overly causal claims with more measured interpretations.

6. Ethical and Data Transparency: The ethics statement has been expanded with sufficient detail on the oral consent procedure, and a clear Data Availability Statement has been added, meeting journal standards.

In summary, the revised manuscript is now methodologically sound, theoretically grounded, and clearly presented. The authors have successfully transformed a manuscript with potential into a robust and publishable research article that makes a valuable contribution to the fields of rural geography, spatial justice, and traditional village studies. The study's proposed three-dimensional model offers a practical, empirically validated tool for assessing justice perceptions in transitioning rural landscapes.

Therefore, I recommend acceptance of the manuscript in its current form.

Decision: Accept

**********

7. PLOS authors have the option to publish the peer review history of their article (what does this mean?). If published, this will include your full peer review and any attached files.

If you choose “no”, your identity will remain anonymous but your review may still be made public.

**Do you want your identity to be public for this peer review?**  For information about this choice, including consent withdrawal, please see our Privacy Policy.

Reviewer #2: **Yes:** Jalil Ahmad

Reviewer #3: **Yes:** Dr. Maria Qayyum

**********

Attachment Submitted filename: Reviwer Comments.docx

# Editor report: Acceptance letter

**DOI:** 10.1371/journal.pone.0352578.r004

**Authors:** Jamil Afzal

PONE-D-25-67060R1

PLOS One

Dear Dr. Deng,

I'm pleased to inform you that your manuscript has been deemed suitable for publication in PLOS One. Congratulations! Your manuscript is now being handed over to our production team.

At this stage, our production department will prepare your paper for publication. This includes ensuring the following:

* All references, tables, and figures are properly cited

* All relevant supporting information is included in the manuscript submission,

* There are no issues that prevent the paper from being properly typeset

You will receive further instructions from the production team, including instructions on how to review your proof when it is ready. Please keep in mind that we are working through a large volume of accepted articles, so please give us a few days to review your paper and let you know the next and final steps.

Lastly, if your institution or institutions have a press office, please let them know about your upcoming paper now to help maximize its impact. If they'll be preparing press materials, please inform our press team within the next 48 hours. Your manuscript will remain under strict press embargo until 2 pm Eastern Time on the date of publication. For more information, please contact onepress@plos.org.

You will receive an invoice from PLOS for your publication fee after your manuscript has reached the completed accept phase. If you receive an email requesting payment before acceptance or for any other service, this may be a phishing scheme. Learn how to identify phishing emails and protect your accounts at https://explore.plos.org/phishing.

If we can help with anything else, please email us at customercare@plos.org.

Thank you for submitting your work to PLOS ONE and supporting open access.

Kind regards,

PLOS ONE Editorial Office Staff

on behalf of

Dr. Jamil Afzal

Academic Editor

PLOS One
